# Merging Biocatalysis and Chemocatalysis in Flow: State‐of‐the‐Art and Future Directions for Sustainable Synthesis

**DOI:** 10.1002/anie.202511930

**Published:** 2026-05-01

**Authors:** Petros Siasiaridis, Matteo Damian, Francesco G. Mutti

**Affiliations:** ^1^ Van 't Hoff Institute for Molecular Sciences HIMS‐Biocat University of Amsterdam Amsterdam The Netherlands

**Keywords:** biocatalysis, chemocatalysis, chemo‐enzymatic cascades, flow chemistry, green chemistry

## Abstract

The growing demand for complex molecules continues to drive innovation in organic synthesis, yet challenges in sustainability, selectivity, scalability, and harsh reaction conditions persist. Enzymes offer exquisite chemo‐, regio‐, and stereoselectivity under mild conditions, while chemocatalysis provides robust and versatile reactivity. However, integrating these approaches into streamlined processes remains difficult due to incompatible conditions and operational constraints. Continuous flow chemistry offers a promising solution by enabling the efficient combination of biocatalysis and chemocatalysis, while improving atom economy, reaction control, scalability, and energy efficiency. This review highlights key advances up to 2025 in merging enzymatic and chemical steps into streamlined continuous flow cascades. It analyzes examples involving various enzyme classes—hydrolases, oxidoreductases, lyases, transferases, and isomerases—used alongside chemical catalysts. Major challenges such as enzyme immobilization, catalyst leaching, and reactor clogging are discussed, along with innovative solutions. The review also discusses how advanced enzyme engineering and immobilization strategies enhance biocatalyst activity, stability, and compatibility with chemical steps. By outlining recent progress and future directions, this review emphasizes how the integration of biocatalysis, chemocatalysis, and flow chemistry can foster more sustainable and efficient synthetic methodologies, particularly relevant to the pharmaceutical and fine chemical industries.

## Graphical abstract

This review highlights recent advances in integrating biocatalysis and chemocatalysis in continuous flow to create streamlined, sustainable processes. It examines chemo‐enzymatic cascades combining at least one enzymatic and one chemical step, discusses challenges such as enzyme immobilization, leaching, and reactor clogging, and presents solutions like enzyme engineering, reactor design, and automation that enhance compatibility and efficiency.

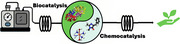

## 1 Introduction

The synthesis of complex organic molecules is a cornerstone of modern chemistry, essential to produce a plethora of important products such as pharmaceuticals, agrochemicals, polymers, and high‐tech materials [1, 2]. Traditionally, these syntheses rely on classical organic chemistry methods, which often involve many steps, harsh reaction conditions, and the use of hazardous reagents [3, 4, 5, 6, 7]. Despite their effectiveness, these methods exhibit significant limitations, particularly in chemo‐, regio‐, and stereoselective transformations. Such constraints arise from the limited selectivity of conventional reagents and catalysts, the need for extensive purification, and the sensitivity of the reactions.

Enzymes offer a promising solution to these challenges [8, 9]. These biological catalysts have emerged as powerful “agents” capable of overcoming many limitations associated with classical organic synthesis. They provide exceptional selectivity, enabling various chemo‐, regio‐, and stereoselective reactions under mild conditions [10, 11]. Operating in aqueous environments at ambient temperatures and pressures, enzymes reduce the need for toxic solvents and harsh reaction conditions. This selectivity and operational simplicity make enzymes particularly valuable for the synthesis of chiral molecules, which are crucial, especially in the pharmaceutical industry, due to their desired specific biological activities [12, 13, 14].

However, the industrial application of enzymes poses certain challenges, especially in scaling up enzymatic processes [15, 16]. Biocatalyzed reactions can sometimes be slow, and the enzymes’ stability and cost can limit utilization at industrial scale. Optimizing these processes is complex, as each distinct enzyme has specific requirements regarding temperatures, pH, and substrate concentration, along with potential incompatibility issues with other reagents. Furthermore, enzyme stability over time is a critical concern as enzymes can denature or aggregate [17, 18], leading to expensive downstream processing and purification requirements. Additionally, using water as a solvent in large reactors can also introduce issues such as insufficient substrate solubility, low product yields, high energy demands for heating and mixing, different physical properties of the mixture, and costly post‐synthetic treatment processes [19].

Flow chemistry offers a way to overcome these challenges by enabling the continuous processing of chemicals through reactors. Continuous flow systems enhance reactivity and selectivity, offer better control over reaction conditions, improve heat and mass transfer, and allow for the integration of multiple reaction steps in a single process, known as telescoping [20, 21, 22]. This makes flow chemistry an ideal platform for implementing chemo‐enzymatic synthesis upon enzyme immobilization [23, 24, 25], as it allows the precision of enzymatic catalysis to be combined with the efficiency and scalability of continuous flow processes [26, 27, 28, 29, 30, 31, 32, 33, 34, 35, 36, 37, 38]. Moreover, flow chemistry aligns with regulatory expectations from both the U.S. Food and Drug Administration (FDA) and the European Medicines Agency (EMA) by supporting consistent product quality and facilitating access to larger production volumes through numbering‐up [39]. Finally, these systems can provide significant advantages for cofactor‐dependent enzymatic reactions, by enabling more efficient recovery of the cofactor through catch‐and‐release strategies, which can be easily and readily incorporated in many chemo‐enzymatic processes [40, 41, 42, 43]. Nonetheless, cofactor‐dependent enzymes still face limitations due to recycling issues, while the turnover numbers of many batch processes can be improved by transitioning to flow reactors. Advances in protein engineering [44, 45, 46, 47, 48, 49, 50, 51, 52], and cofactor and site‐specific immobilization strategies can enhance enzyme stability and functionality in flow systems, addressing these challenges [53, 54].

In this review, we explore the realm of chemo‐enzymatic synthesis in flow by presenting literature examples grouped by enzyme class (i.e., hydrolases, oxidoreductases, lyases, transferases, and isomerases), in which the combination of enzymes and chemocatalysts has proven successful under continuous flow conditions. After reviewing the literature, we discuss the current challenges and future opportunities of these systems while providing our perspective on the importance of implementing such processes.

### 1.1 Hydrolases

Hydrolases are by far the most employed biocatalysts in the literature related to chemo‐enzymatic applications in continuous flow. This class of enzymes is usually utilized with water as reagent and solvent for hydrolysis reactions resulting, in most cases, in breaking down large molecules into smaller ones. Certain hydrolases can also function in nonaqueous environments to catalyze the formation of, for example, ester and amide bonds [55]. Within the broader hydrolase family, lipases, proteases (amidases), and esterases are of particular importance. These enzymes are utilized for regioselective or stereoselective hydrolytic biotransformations, enabling the production of intermediates for pharmaceuticals, pesticides, and chiral precursors for asymmetric synthesis. Lipases, in particular, are widely used to hydrolyze triglycerides and fats, into fatty acids and glycerol. Among them, *Candida antarctica* lipase B (CalB) stands out due to its robustness and has been extensively utilized to produce optically active alcohols, acids, esters, and lactones through kinetic resolution [56].

**SCHEME 1:**
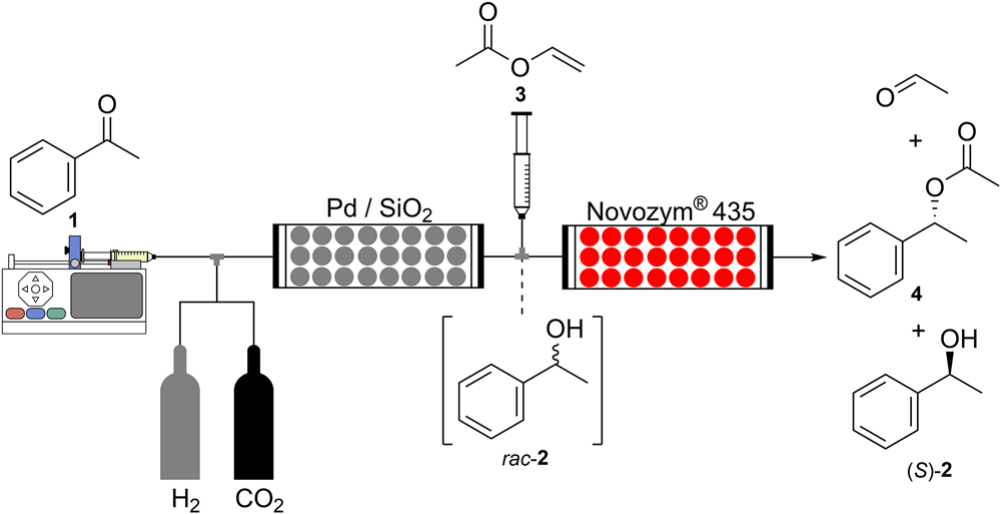
Chemoenzymatic synthesis of enantiopure phenylethanol via Pd‐catalyzed hydrogenation of acetophenone followed by kinetic resolution catalyzed by immobilized CalB under continuous supercritical flow conditions.

Hobbs et al. presented one of the first examples of combining a chemical and an enzymatic step under continuous flow conditions (Scheme 1) [55]. This work also demonstrated for the first time the feasibility of combining biocatalytic and metal‐catalyzed reactions in multi‐step continuous flow processes using supercritical CO_2_. The process consists of the Pd‐catalyzed continuous hydrogenation of acetophenone (**1**), followed by the kinetic resolution of the racemic 1‐phenylethanol intermediate (**2**) in the presence of vinyl acetate (**3**), catalyzed by Novozym 435 (a commercial immobilized CalB). The process begins with the delivery of H_2_ (H_2_:acetophenone, molar ratio = 4:1) in CO_2_ (flow rate of 1 mL min^−1^) at 100 bar pressure, under controlled dosages in a mixer. Acetophenone (**1**) is introduced with a flow rate of 0.1 mL min^−1^ using an external HPLC pump and is mixed with the gases before passing through the first reactor, which contains 2% Pd catalyst supported on silica/alumina. The resulting intermediate (**2**) is then combined with compound **3** and fed into the enzyme reactor, maintained at 50°C, containing 250 mg of CalB to perform the kinetic resolution. The study reported a maximum conversion of 71–74% for the hydrogenation step, followed by recovery of 30–35% of acetylated product (**4**) in >99% enantiomeric excess. Unfortunately, no stability test of the entire reactor was reported, leaving it unclear whether the system remains stable over multiple days.

**SCHEME 2:**
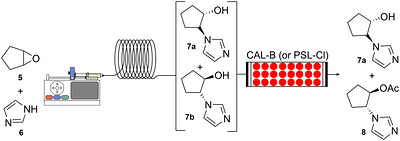
Schematic setup for the epoxide ring‐opening reaction and kinetic resolution under flow conditions.

Another early example of a system that combined a chemical step with an enzymatic step under continuous flow conditions was by Porcar et al. in 2012 (Scheme 2) [57]. Although their study did not report the telescoping of the two steps in a single operation, meaning the transformations were conducted separately, it is noteworthy as it demonstrated the feasibility of the combination without significant modifications. The first part of the process involved the ring‐opening reaction of the epoxides (**5**) (either five‐ or six‐membered) using imidazole (**6**) in dry THF as the nucleophile. The mixture was passed through a tubular reactor heated by microwave irradiation or conventional heating (17 bar and 140°C) at varying flow rates to yield the corresponding (±) *trans*‐2‐(1H‐imidazol‐1‐yl)cyclopentanol (enantiomers **7a** and **7b**) or the analogous cyclohexanol enantiomers. Afterwards, the solvent was concentrated, and the resulting solution was purified by flash chromatography on silica gel to isolate the racemic alcohol as a white solid. Subsequently, a solution of the racemic alcohol and vinyl acetate in dry THF was pumped under an inert atmosphere through a packed column containing the biocatalyst. Two different purified enzymes were tested, at 45°C under 40 bar pressure, namely, the *Candida antarctica* lipase B (CalB) and the *Pseudomonas cepacia* lipase (PSL‐C I, also known as *Burkholderia cepacia* lipase). After purification through flash chromatography, the corresponding optically enriched acetates ((*R,R*)‐*trans*‐2‐(1H‐imidazol‐1‐yl)cyclopentylacetate or cyclohexylacetate analogue) (**8**) and alcohols ((*S,S*)‐*trans*‐2‐(1H‐imidazol‐1‐yl)cyclopentanol or cyclohexanol analogue) (**7a**) were isolated in pure form. Even though, as mentioned, the steps were not conducted continuously, their work is particularly interesting as it employs microwave heating and biocatalysts to efficiently produce enantiopure imidazoles. These molecules find applications in the preparation of chiral ionic liquids (CILs), asymmetric phase transfer catalysts, and as precursors for stationary phases in gas and liquid chromatography. Interestingly, the system demonstrated high stability, carrying out the kinetic resolution of the mixture of **7a** and **7b** for 11 days without any loss of biocatalytic activity or enantioselectivity.

**SCHEME 3:**
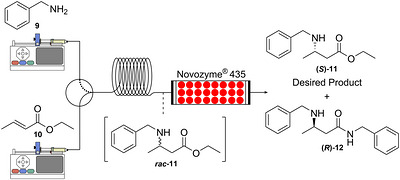
Solvent‐free chemo‐enzymatic reaction sequence for the synthesis of β‐amino ester.

In 2013, Strompen et al., published one of the first studies on chemo‐enzymatic syntheses involving lipases for the enantioselective synthesis of a β‐amino acid ester and β‐amino amides in flow (Scheme 3) [58]. This work exemplifies an early instance of a coupled chemo‐enzymatic process in flow, and by eliminating the use of solvents, it presents a more atom‐efficient and sustainable method for producing a β‐amino ester and β‐amino amides. When performed in a CSTR, the longer reaction times in batch compared to flow favor the spontaneous nonselective aminolysis of *rac*‐**11** to yield byproduct *rac*‐**12**, resulting in a reduced yield of desired (*S*)‐**11**. However, this uncontrolled chemical aminolysis does not occur when using a plug‐flow reactor due to the reduced residence time. The flow process consisted of two steps: a solvent‐free thermal Aza‐Michael addition followed by an enzymatic kinetic resolution (KR). The process began with two pumps containing benzylamine (**9**) and *trans*‐ethyl crotonate (**10**) that were mixed before entering the tubular reactor pre‐heated at 80°C. This step resulted in a racemic mixture of the intermediate product (*rac*‐**11**) at 92% conversion. The resulting mixture, containing some of the unreacted benzylamine (**9**), was directed toward the PBR for the biocatalytic aminolysis. For this transformation, purified *Candida antarctica* lipase B (often abbreviated as CalB, or frequently reported using the commercial brand Novozym 435) was selected and immobilized on a resin (Lewatit VP OC 1600). The borosilicate glass reactor packed with 6 g of Novozym 435 was maintained at 60°C while the mixture was pumped through at a flow rate of 0.4 mL·min^−1^ yielding a conversion of 59% toward the desired product. Overall, the continuous production of (*S*)‐ethyl 3‐(benzylamino)‐butanoate ((*S*)‐**11**) was accomplished, with a total conversion of 54% and an excellent enantiomeric excess of 98%. The process could be operated for more than 88 h without showing any significant loss of activity, resulting in a space time yield of 128 g·L^−1^·h^−1^.

**SCHEME 4:**
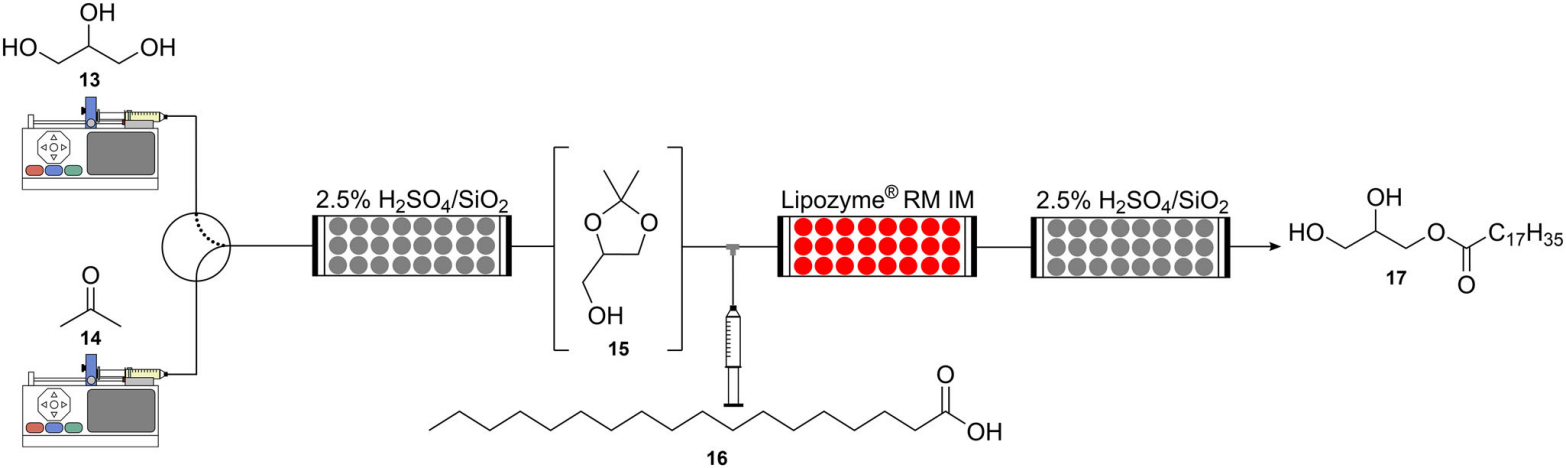
Chemoenzymatic route to 1‐monoacylglycerol (**17**).

In the same year, Itabaiana Jr. et al. reported a three‐step chemoenzymatic continuous flow cascade for the synthesis of 1‐monoacylglycerol (**17**) from biodiesel‐derived glycerol (Scheme 4) [59]. Despite the low overall yield (15%) and the absence of enzymatic selectivity, this flow process represented an interesting attempt at the valorization of renewable resources, such as glycerol derived from the biodiesel industry and fatty acid residues from oil refining processes. Starting from glycerol (**13**) and acetone (**14**) in a 6:1 molar ratio, the mixture was pumped into the first reactor containing 2.5% w/v H_2_SO_4_ over a functionalized silica gel as the catalyst, with a flow rate of 0.2 mL·min^−1^ at 60°C. This step yielded the desired 1,2‐O‐isopropylideneglycerol (**15**), the protected intermediate required because the lipase was not selective for primary alcohols. Next, the protected glycerol was directed toward a packed‐bed reactor (PBR) containing the biocatalyst for esterification of the intermediate. The researchers used Lipozyme RM IM, a commercial lipase preparation of *Rhizomucor miehei*, immobilized on a microporous anion‐exchange resin. The enzyme loading was 24.3 mg·g^−1^_support_, with a total amount of 420 mg. A pump was used to add stearic acid (**16**) to the stream containing the protected glycerol intermediate before it entered the reactor, achieving a productivity of 518 g·h^−1^·g^−1^_immobilized enzyme_. The final step involved acid‐catalyzed deprotection of the 1‐monoacylglycerol ketal. The mixture was again directed through a 2.5% w/v H_2_SO_4_/SiO_2_ column at a flow rate of 0.2 mL·min^−1^, where the product formation was maximized with no byproducts detected. Notably, the final product did not require further purification or laborious isolation, as spontaneous crystallization occurred at 50°C in the final solution. This study demonstrates a direct synthesis of 1‐monoacylglycerol (**17**) without any solvent change. Stable conversion was maintained for the first 18 cycles (defined as the passage of one column volume), after which it decreased markedly. This decrease of conversion was attributed to enzyme leaching. Although this chemoenzymatic sequence proceeded without enantioselectivity—since the applied commercial lipases convert both enantiomers of **15**—a kinetic resolution (KR) of *rac*‐**15** to yield enantiopure **17** would be conceivable if a selective hydrolase were identified or engineered.

**SCHEME 5:**
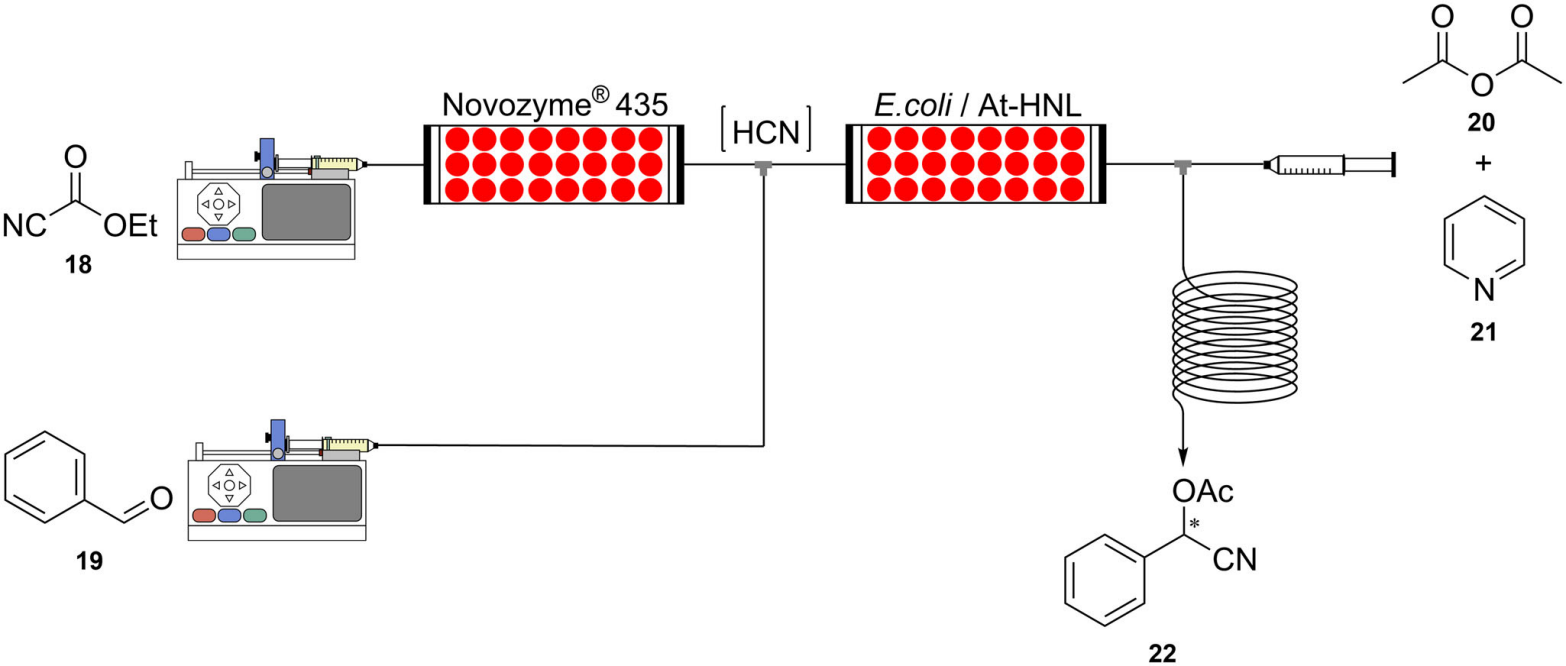
Three‐step chemo‐enzymatic cascade for the synthesis of O‐protected chiral cyanohydrins (**22**).

Two years later, Brahma et al. proposed an orthogonal biocatalytic approach for the safe generation and use of HCN in a multistep continuous flow preparation of chiral O‐acetylcyanohydrins (**22**) (Scheme 5) [60]. This work demonstrates the advantages of continuous flow processes when dealing with dangerous reagents such as HCN, as well as its effective combination with whole cells biocatalysis and immobilized enzymes for carrying out complex transformations. In this article, the scientists exploited the CalB lipase alongside a hydroxynitrile lyase (HNL) to access chiral cyanohydrins from aldehydes or ketones and HCN. The system consisted of three steps: two consecutive enzymatic ones under 5 bar pressure, followed by an in‐line chemical acetylation that served as protection. The process began with the pumping of the ethyl cyanoformate (**18**) (ECF) solution in methyl tert‐butyl ether (MTBE) through the first PBR containing 277 mg of the CalB lipase immobilized on acrylic resin (Novozym 435) at a flow rate of 0.04 mL·min^−1^. There, the hydrolysis of ECF produced HCN in situ. The HCN stream was mixed with benzaldehyde (**19**), using another pump, in micro‐aqueous MTBE at a T junction at a flow rate of 0.04 mL·min^−1^, and the mixture was directed to the next PBR. For this step, the biocatalyst used was *E. coli* BL21(DE3) whole cells overexpressing a wild‐type hydroxynitrile lyase (AtHNL), which catalyzed the addition of HCN to aldehydes. The resulting cyanohydrins were then protected due to their instability at neutral or alkaline pH at room temperature. To perform the protection, a third pump was utilized, adding Ac_2_O (**20**) and pyridine (**21**) to the stream, which was finally directed toward a 2 mL PTFE coil reactor to obtain the protected cyanohydrins. The continuous flow system was robust and could be applied to a broad range of electron‐rich and electron‐poor O‐acetylcyanohydrins, showcasing good conversions in the range of 75–99% and high stereo‐control (40–98%). Continuous flow was implemented to overcome safety and operational limitations of batch biocatalysis. In situ HCN generation via CalB‐catalyzed ECF hydrolysis minimizes handling of toxic HCN or KCN, while precise control of residence time, temperature, and biocatalyst loading ensures reproducibility. The CalB reactor maintained high conversion over several hours, whereas in batch the enzyme activity dropped from 99% to 70% after four cycles. Flow also enables seamless telescoping of ECF hydrolysis, HCN addition to aldehydes, and in‐line acetylation, and the micro‐aqueous setup minimizes HCN accumulation, allows pressurization, and supports scalable, regulatory‐compliant operation.

**SCHEME 6:**
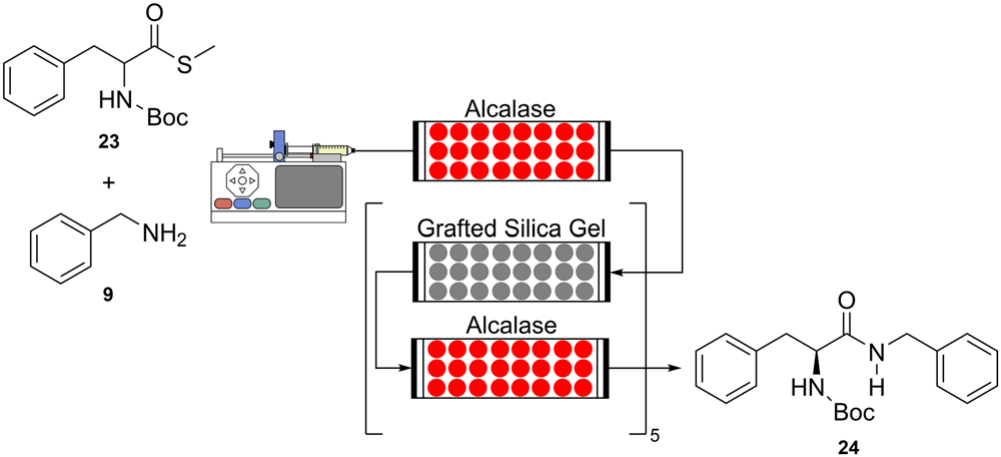
DKR of racemic N‐Boc‐phenylalanine ethyl thioester in continuous flow.

In 2016, Falus et al. described the catalyzed dynamic kinetic resolution (DKR) of N‐Boc‐phenylalanine ethyl thioester as substrate (**23**), using benzylamine (**9**) as nucleophile, in a continuous flow cascade reactor system (Scheme 6) [61]. In general, this work provides a potential platform for combining various kinetic resolution and racemization steps that require distinct conditions, such as optimal temperatures. The DKR process consisted of an alternating cascade of packed‐bed columns containing the enzymatic or racemization catalysts at different temperatures. To begin, a solution of the racemic substrate, benzylamine (**9**) and 1,8‐diazabicyclo[5.4.0]undec‐7‐ene (DBU) in *tert*‐amyl alcohol was pumped through the first stainless steel column containing the biocatalyst, which was externally heated at 50°C at a flow rate of 0.2 mL·min^−1^. The enzyme used was a purified alcalase (Subtilisin A), immobilized on ethyl‐grafted microporous silica gel (Dv250‐Et). This step resulted in the kinetic resolution of the substrate, yielding only one of the enantiomeric products, while the other one remained intact. Remarkably, this step was particularly robust, as high productivity and enantiomer selectivity were maintained even after 5 continuous days of operation. The mixture of the product and the residual enantiomer of the ethyl thioester was then directed into the next packed‐bed column, which contained only the ethyl‐grafted silica gel. There, racemization occurred under optimal conditions using DBU at 150°C. This cycle was repeated five more times (a total of 11 columns were employed) to reach a steady‐state in approximately three hours. HPLC analysis showed that the continuous flow DKR achieved a conversion of around 79% with excellent enantioselectivity (98%), approaching the limit of kinetic control. Finally, the product (**24**) was easily isolated from the effluent in a good yield (71%) as a white solid. The overall system achieved a volumetric productivity of 8 g·L^−1^·h^−1^.

**SCHEME 7:**
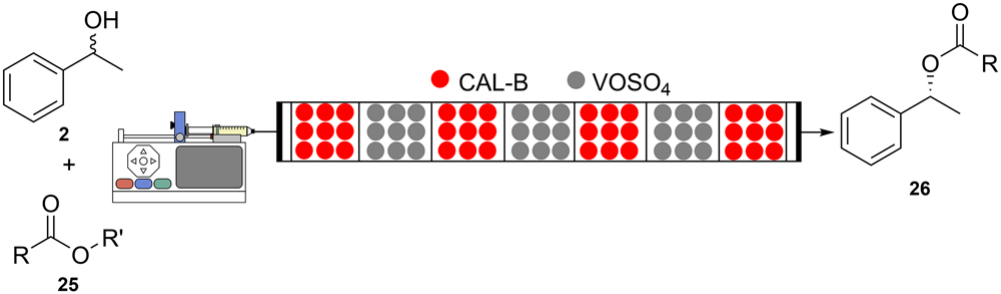
Setup of the DKR of racemic 1‐phenylethanol in continuous flow conditions.

Another application combining a racemization catalyst and an enzyme to achieve DKR was showcased by de Miranda et al. in 2017 (Scheme 7) [62]. The system consisted of two distinct steps for the DKR of racemic 1‐phenylethanol (**2**) in a continuous flow PBR with alternating layers of immobilized enzyme and racemization catalyst. The substrate (**2**), along with different acyl donors (**25**) (ethyl acetate, vinyl acetate, vinyl decanoate, etc.) in toluene, were pumped through the PBR, which contained four layers of purified CalB immobilized on a resin (Novozym 435) and three layers of vanadyl sulfate (VOSO_4_) that promotes the racemization of benzylic alcohols. These layers were arranged in a stacked configuration, separated by thin cotton films, which were crucial in preventing any incompatibility issues between the two catalysts. The temperature was set at 70°C to ensure optimal activity of both the enzyme and the chemo‐catalyst. Using vinyl acetate as the acyl donor resulted in a conversion of 92% with a 99% enantiomeric excess at a residence time of 5.2 min, achieving a productivity of approximately 1 g·h^−1^. However, longer residence times led to lower yield and enantiomeric excess, due to the inhibition of the racemization catalyst by the acyl donor. On the other hand, when using vinyl decanoate, the (*R*)‐phenylethyl decanoate product was obtained with 90% ee albeit at a lower yield of 82%, continuously for 2 h. Various parameters such as residence time, acyl donor concentration, flow rates, and even recirculation techniques were tested to determine the optimal conditions showcasing the versatility of this system. However, the authors did not assess the operational lifetime of the reactor.

**SCHEME 8:**
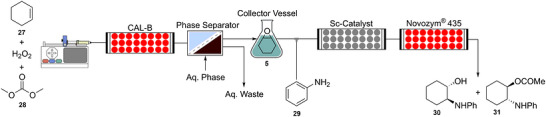
Setup of the three‐step continuous flow synthesis of an enantiopure 1,2‐amino alcohol (**30**) and 1,2‐aminocarbonate (**31**).

A year later, Porcar et al. published a paper on utilizing dimethyl carbonate (**28**) (DMC) as a noninnocent yet benign solvent for the three‐step continuous flow synthesis of amino alcohols (Scheme 8) [63]. This noteworthy work demonstrated the use of DMC that also functioned as a reagent in the first step, resulting in the formation of fewer impurities and simpler side products, such as CO_2_ and MeOH. As a result, less complex and costly purification techniques were required, making the whole process more viable and sustainable. Starting with the first step, the epoxidation of cyclohexene (**27**), the purified CalB lipase was immobilized on polymer‐supported ionic liquids (SILLPS) containing 1‐decyl‐2‐methylimidazolium cations covalently attached to a polystyrene divinylbenzene porous matrix, with NTf_2_^−^ as the counterion, thus creating the biocatalyst CalB‐SILLP‐dec‐NTf_2_. Here, alongside cyclohexene and hydrogen peroxide, DMC was added and used both as a reagent and a solvent. The mixture was then directed toward the first PBR, containing 1 g of the above‐mentioned enzyme at a flow rate of 10 µL·min^−1^ at 45°C, resulting in the production of the corresponding epoxide with a yield of around 90% for more than 95 h continuously. The next step was the synthesis of the racemic 1,2‐amino‐alcohol through the epoxide ring opening in the presence of aniline (**29**). An external pump introduced aniline into the epoxide stream at a flow rate of 10 µL·min^−1^, and the mixture was directed toward the next PBR containing the polymer immobilized scandium catalyst, the specific details of which were not disclosed in the paper. This catalyst provided a robust and stable performance for 97 h of continuous operation, achieving complete conversion of the starting material. Initially, the researchers attempted to combine these first two steps but failed due to the excess of H_2_O_2_ interfering with the second step. The authors’ solution was to implement a liquid‐liquid separator to ensure the removal of H_2_O_2_ before the second reaction. For the final step, they examined the kinetic resolution (KR) of these alcohols by enzymatic means. A PBR packed with CalB immobilized on a resin (Novozym 435) was used to achieve the transesterification, employing DMC as both the solvent and acyl donor. This approach achieved 48% conversion into the desired chiral carbonate (**31**), with an excellent enantioselectivity of >99%, while the entire system operated continuously for 27 h without any significant loss in activity or selectivity and without requiring any intermediate purification steps.

**SCHEME 9:**
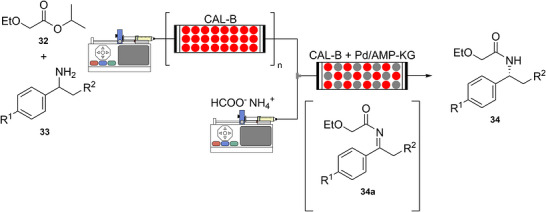
Schematic representation of the DKR of benzylic amines (**33**) in continuous flow.

Yet, another example of a chemo‐enzymatic system for the dynamic kinetic resolution under continuous flow was presented by Farkas et al. in 2018 (Scheme 9) [64]. The process starts with a mixture of a racemic benzylic amine (**33**) and isopropyl 2‐ethoxyacetate (**32**) as the solvent and acylating agent, which is directed to the first PBR containing a sol‐gel matrix immobilized with purified CalB as the biocatalyst for the resolution step. The outflow of this reactor is mixed in a T junction with ammonium formate, which pre‐activates the Pd‐catalyst and suppresses some undesired side reactions during the metal‐catalyzed step. The solvent used was a 1:1 (v/v) mixture of 2‐methyl‐2‐butanol and toluene to enhance the solubility of the more hydrophilic amines. The temperature throughout was maintained at 60°C, and the flow rate of all pumps was set at 5 µL·min^−1^. This solution is then directed toward the next column containing both the biocatalysts and the racemization catalyst, which is palladium on 3‐aminopropyl‐functionalized silica. The role of the palladium, preactivated with a flow of ammonium formate solution, is to racemize the unreacted enantiomer to provide a continuous supply of substrate for the enzyme. This procedure proceeds via the imine intermediate **34a**. For the metal‐catalyzed racemization process, multiple side reactions can produce side products such as further imines, secondary amines, hydrocarbons, and ketones. These undesired side reactions are suppressed by the additional dihydrogen and ammonia sources, in this case ammonium formate. The result is the production of the amide (**34**) in high isolated yield (96%) with an excellent enantiomeric excess (>99%). However, these numbers fluctuate based on the starting benzylic amine, and in some cases, more than one PBR, containing the biocatalyst, was used in series to achieve satisfactory conversion. The process was evaluated using aliphatic and arylaliphatic amines lacking additional functional groups; therefore, broad substrate applicability was not demonstrated. Despite this limitation, the system was stable, demonstrating performance for at least two days and allowing for the efficient and consistent production of enantiomerically pure amides from racemic mixtures of valuable benzylic amines.

**SCHEME 10:**
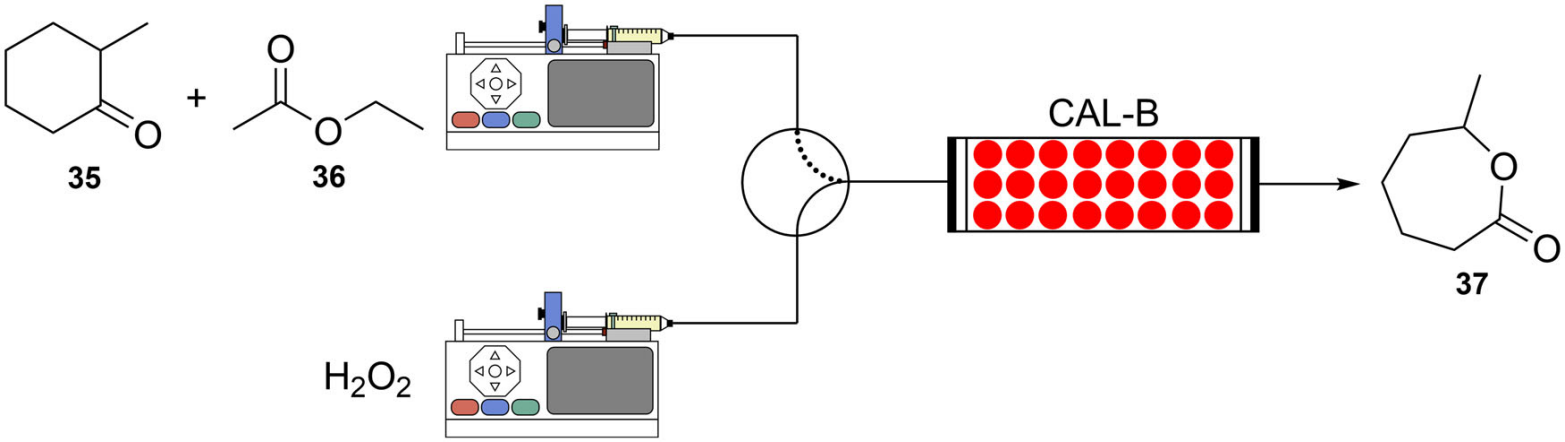
Schematic depiction of continuous‐flow chemo‐enzymatic Baeyer–Villiger oxidation.

In 2019, Szelwicka et al. designed a continuous flow chemo‐enzymatic Baeyer−Villiger oxidation using a CalB lipase immobilized via physical adsorption onto commercially available multi‐walled carbon nanotubes (CalB Nanocyl NC7000 MWCNTs) (Scheme 10) [65]. A notable feature is the versatility of this system, which provides a potentially scalable method for synthesizing lactones that are useful in the fine chemical industry [66]. In this process, the CalB lipase performs the catalytically promiscuous oxidation of ethyl acetate with H_2_O_2_ in order to generate the peracid which then oxidizes the cyclic ketones to lactones. The system was tandem‐like, consisting of two pumps coming together through a three‐way valve in a mixer before entering the column reactor. One pump contained the organic phase, consisting of a mixture of the substrate (2‐methylcyclohexanone (**35**) alongside ethyl acetate (**36**) as the peracid precursor and solvent. The second pump contained the aqueous solution of H_2_O_2_. The presence of the mixer allowed for the in situ generation of peracid, which is the ultimate reagent, through the reaction between hydrogen peroxide and ethyl acetate. Then, the solution was directed to the column reactor which was densely packed with 0.5 g of CalB/Nanocyl NC7000. The optimal residence time for achieving 87% conversion of the ketone to 6‐methyl‐ε‐caprolactone (**37**) was found to be 5 min, while the nano‐biocatalyst was stable even after 8 h of continuous running. Even though a notable decrease in the conversion rate was measured after 24 h due to enzyme deactivation, it was possible to reuse the support (Nanocyl NC7000 MWCNTs) by burning off the residual protein in the furnace and reloading a fresh aliquot of the enzyme. Overall, this method exhibited an efficient chemo‐enzymatic oxidation to lactones using an undeniably active and robust catalyst in continuous flow.

**SCHEME 11:**
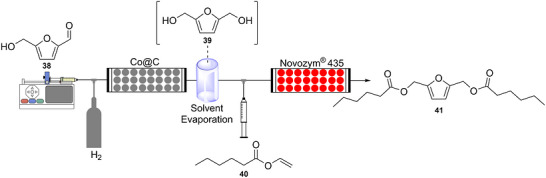
Chemoenzymatic synthesis of 5‐hydroxymethylfurfural(HMF)‐derived plasticizers (**41**).

In 2020, Arias et al. reported the chemoenzymatic synthesis of 5‐hydroxymethylfurfural (HMF)‐derived plasticizers (Scheme 11) [67]. The first step of the process involves the chemical reduction of HMF (**38**) to yield 2,5‐bis(hydroxymethyl)furan (BHMF) (**39**) in a fixed‐bed reactor, using H_2_ (10 bar) and a Co@C catalyst, with methanol as the carrier solvent. The optimal temperature for this step was found to be 90°C, with a flow rate of 12 mL·h^−1^. After methanol removal, the reaction mixture (1.3 wt% of BHMF), along with vinyl hexanoate (**40**, 6 wt%) as the acyl donor in 2‐methyltetrahydrofuran as the solvent, was fed into a second fixed‐bed reactor containing the hydrolase Novozym 435, maintained at 35°C. This step afforded an 88% yield of the corresponding diester product (**41**), with the system operating continuously for 60 h without any observable loss in enzymatic activity. Due to solvent incompatibility, the two catalytic steps were connected via an intermediate step in which the methanol used in the first reaction was removed in a collector through flash evaporation. This allowed the methanol to be recycled for the first step, while the reaction mixture could proceed to the second reactor without interruption.

**SCHEME 12:**
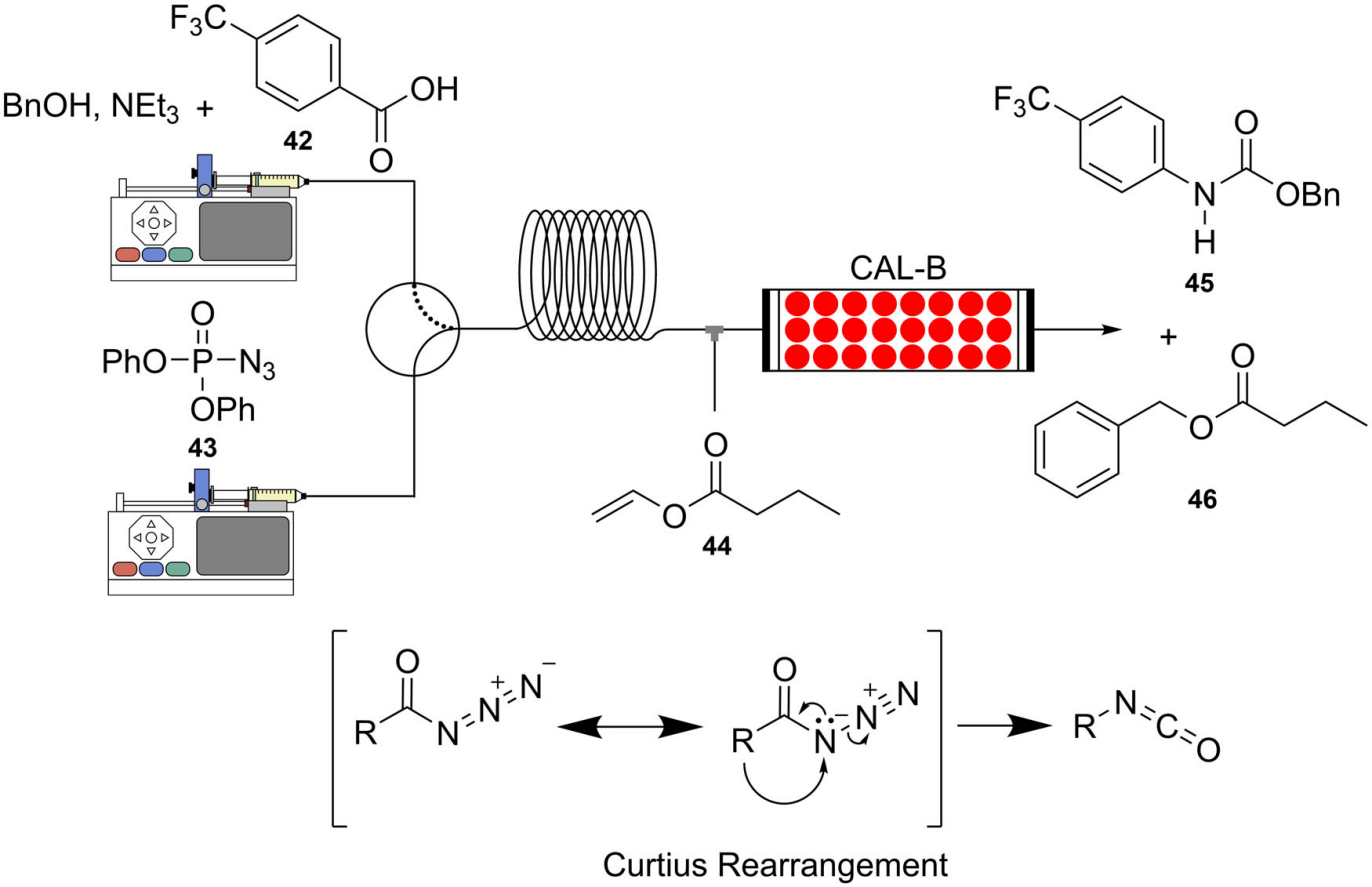
Flow setup involving the tandem Curtius rearrangement and CalB‐mediated BnOH tagging.

A different use of the lipase CalB was showcased by Baumann et al. in 2020 in a tandem continuous flow process (Scheme 12) [68]. This work brings forward an alternative use of enzymes in chemo‐enzymatic continuous flow applications since the lipase was not utilized to achieve a certain transformation toward the desired product; rather, it was employed to tag the impurities so that they can be easily purged during the isolation process. This approach possesses the potential for many more diverse uses of enzymes in a synthetic pathway. This system consisted of two steps: first, the Curtius rearrangement of the substrate; and second, the chemo‐selective tagging of the residual reagent, resulting in a more facile purification. The process starts with two pumps, both at a flow rate of 0.5 mL·min^−1^, with the first pumping 4‐(trifluoromethyl)benzoic acid (**42**), triethylamine, and benzyl alcohol in toluene, while the second one was used for the pumping of DPPA (**43**) in toluene. The two solutions were mixed in a T‐piece and directed into the coil reactor with a residence time of 30 min at 120°C and 100 psi pressure, using a back‐pressure regulator. Then, a third pump introduced vinyl butyrate (**44**) at a flow rate of 0.5 mL·min^−1^ via a second T‐piece into the stream exiting the coil reactor. The resulting mixture was directed to the packed column containing 3 g of the purified CalB lipase immobilized on an acrylic resin. After a residence time of ca. 5 min, the residual benzyl alcohol was converted to benzyl butyrate (**46**), while the Cbz‐carbamate (**45**) from the Curtius rearrangement remained untouched. The reaction effluent was collected and, after solvent evaporation, was extracted with EtOAc/water. Subsequent crystallization from heptane afforded the carbamate as a white crystalline solid in 83% isolated yield and with a throughput of 6.6 g·h^−1^. The reactor containing CAL‐B proved stable over five reaction cycles (30 min each), although its long‐term operational stability was not further investigated.

**SCHEME 13:**
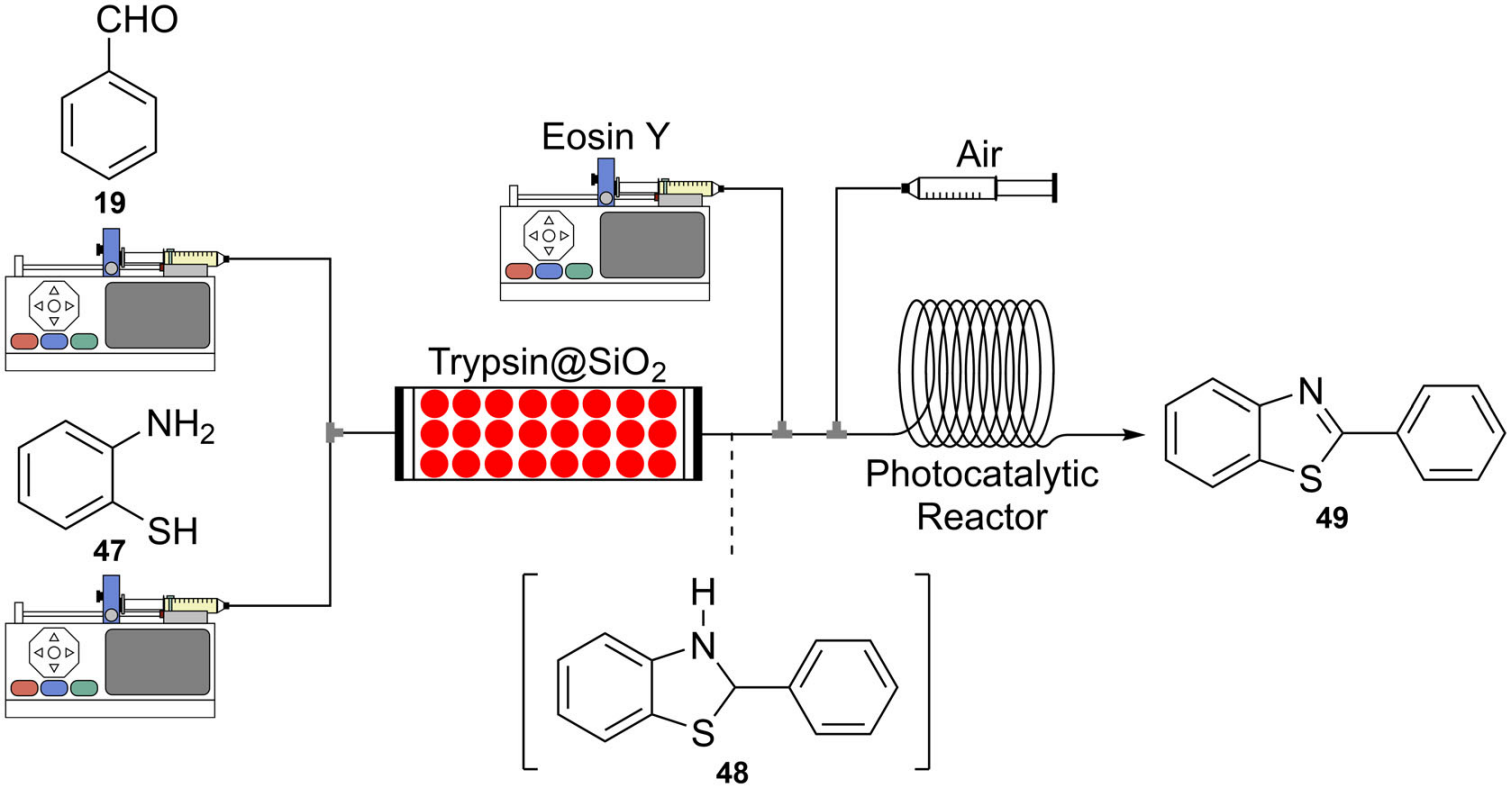
Combination of biocatalysis and photocatalysis in the continuous microflow production of 2‐phenylbenzothiazole (**49**).

In 2021, Wang et al. published a paper on the chemoenzymatic production of 2‐phenylbenzothiazole (2‐PBZ, **49**) (Scheme 13) [69]. They proposed a novel method that combines biocatalysis and photocatalysis in a continuous microflow setup. This work demonstrates how combining biocatalysis and photocatalysis can overcome cross‐inhibition issues between bio‐ and photocatalytic steps, allow online enzyme reuse, ensure uniform light distribution to prevent product decomposition, and ultimately improve overall efficiency, thereby laying the foundation for a more efficient industrial production of 2‐PBZ. The process begins by pumping 2‐aminothiophenol (**47**) and benzaldehyde (**19**) at a constant temperature of 25°C—maintained using a water bath—first into a micromixer and then into a micro‐packed bed reactor (micro‐PBR) containing trypsin hydrolase. The enzyme was immobilized by grafting it onto amino‐functionalized silica particles and catalyzed the condensation reaction between the two substrates. This is another example of the catalytic promiscuity of a hydrolase, which is used for the formation of carbon–nitrogen and carbon–sulfur bonds. The outflow, containing the intermediate (**48**), was then mixed with Eosin Y (as the photosensitizer) and air (serving as the oxygen source for photosensitizer regeneration) and directed into a photocatalytic reactor, where the chemical transformation to 2‐phenylbenzothiazole (**49**) occurred under blue light irradiation. The biocatalytic step required a residence time of 2.6 min, resulting in a space‐time yield 2.3 times higher than that achieved under batch conditions (3559 mmol g^−^^1^ enzyme h^−^^1^ L^−^^1^ vs. 1526 mmol g^−^^1^ enzyme h^−^^1^ L^−^^1^). The photocatalytic step required only 2 min to achieve a yield greater than 99%. The process operated continuously for 300 min, maintaining 93.7% of the total catalytic activity. Notably, no metal catalysts were used, and the reaction conditions were mild. By re‐using the enzyme and harnessing light, with a total residence time of just 4.6 min, the authors achieved an efficient and high‐yielding continuous synthesis of 2‐phenylbenzothiazole. Finally, the authors discussed the potential for scale‐up by numbering up the microflow reactors or integrating them into larger systems, paving the way for large‐scale automated synthesis.

**SCHEME 14:**
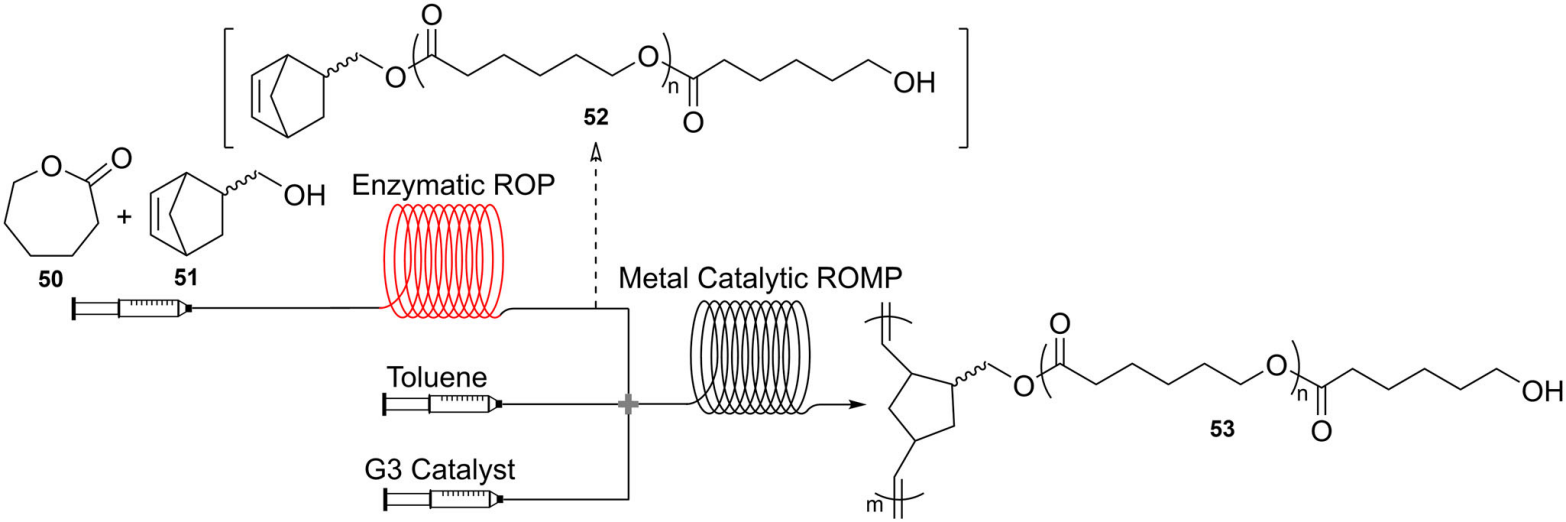
Schematic microreactor setup for the continuous flow chemo‐enzymatic ROP‐ROMP platform.

Two years later, Chen et al., published a paper on microreactor‐based chemo‐enzymatic ring‐opening polymerization (ROP) and metal‐catalytic ring‐opening metathesis polymerization (ROMP) as a platform for continuous flow synthesis of bottlebrush polymers (Scheme 14) [70]. In this work, the authors demonstrated that using microflow technology yields undeniable benefits in the “graft‐through” method of synthesizing bottlebrush polymers. More specifically, higher conversions, easier operation of the reactor due to milder conditions, and even better‐defined structures were obtained, justifying the use of continuous flow systems. In the first step, a norbornene alcohol (**51**) (NB‐OH) acts as the initiator of the ROP of ε‐caprolactone (**50**) (CL) to prepare NB‐terminated poly(ε‐caprolactone) (**52**) (PCL) as the macromonomer, being a part of the final bottlebrush polymer. A mixture of **50** and **51** in toluene was directed into a packed tubular reactor containing the commercially available CalB enzyme immobilized on a resin (Novozym 435) with a residence time of 10 min at 60°C with a flow rate of 0.3 mL·min^−1^, yielding the product at around 92% conversion. The desired product from this reaction is the macromonomer with norbornene as the end group while the byproduct is the poly‐caprolactone without the norbornene at the end, which occurs due to traces of water in the microreactor. For the second step, the macromonomer solution, diluted with toluene and mixed with the metal‐complex ROMP catalyst (Grubbs third generation catalyst, G3), was directed into a PTFE tubular microreactor to produce the desired PNB‐g‐PCL bottlebrush polymer (**53**). For certain starting norbornene alcohols, the produced macromonomer reached a conversion of around 99% in ROMP with a residence time of 400 s. Through fine‐tuning of the reaction conditions, different bottlebrush polymers were synthesized in less than 35 min while the same procedure in batch takes from 120 to 200 min, which is three to five times longer. Moreover, via a microreactor technology, the authors managed to ensure improved control of molecular weight and dispersity. Unfortunately, the authors did not provide any information on the operational stability of the system over extended use.

**SCHEME 15:**
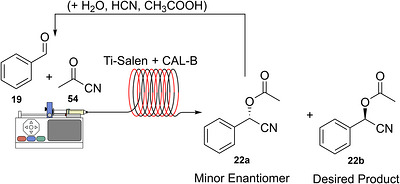
Setup of the cascade reaction for the synthesis of chiral O‐acylated cyanohydrin (**22b**).

In 2022, Zhang et al. published a paper on Pickering emulsion droplet‐based biomimetic microreactors for continuous flow cascade reactions (Scheme 15) [71]. Their system allows the co‐localization of distinct catalytically active subcompartments within droplets of a Pickering (or Ramsden) emulsion. This way, cell‐like microreactors are formed and can be packed into column reactors for catalytic purposes under continuous flow. The two chemo‐enzymatic cascade reactions tested were for the synthesis of chiral cyanohydrins. First, to prepare the biomimetic microreactors, the researchers chose a Pickering emulsion‐based strategy as these are capable of compartmentalizing bulk liquids into nanoliter‐scaled droplets. Next, the chosen molecular catalyst [(salen)Ti‐(μ‐O)]_2_ or Ti(Salen) and the enzyme CalB were supported separately on the mesopores of silica nanoparticles to form the two catalytically active subcompartments (SCs). These SCs were then compartmentalized within ionic liquid (IL) droplets. This was a one‐step emulsification process of a biphasic mixture of [BMIM]PF_6_ (IL), *n*‐octane, and the SCs using hydrophobic silica nanospheres as the emulsifier, which finally yielded the IL‐in‐oil droplet microreactors. To test the catalytic performance of their biomimetic microreactors, the researchers attempted to synthesize chiral O‐acylated cyanohydrins (**22a**, **22b**). They chose a droplet‐based continuous flow system in which a solution of reactants, namely benzaldehyde (**19**) and acetyl cyanide (**54**) as the acyl donor, was pumped into the inlet of the column reactor. The reactor contained the Ti(Salen), which catalyzes the formation of the O‐acylated cyanohydrin, as well as the CalB, which improves the enantioselectivity by hydrolyzing the minor undesired enantiomer back to the starting material. The obtained conversion of benzaldehyde reached 81% and the enantiomeric excess for the product was 99% for 80–240 h. The continuous flow system presented a productivity of 0.6 mol h^−1^, which was 14 times higher than the batch process utilizing the same biomimetic microreactors, 30 times higher when no compartmentalization was apparent, and 420 times higher than the direct mixing of the two catalysts. The authors showcased the importance of successfully co‐localizing the two catalysts. This not only allowed them to fine tune the amount and ratio of the subcompartments but also enabled the “entrapment” of the desired liquid medium, which was proven to be able to bind the reactants inside the microreactor.

**SCHEME 16:**
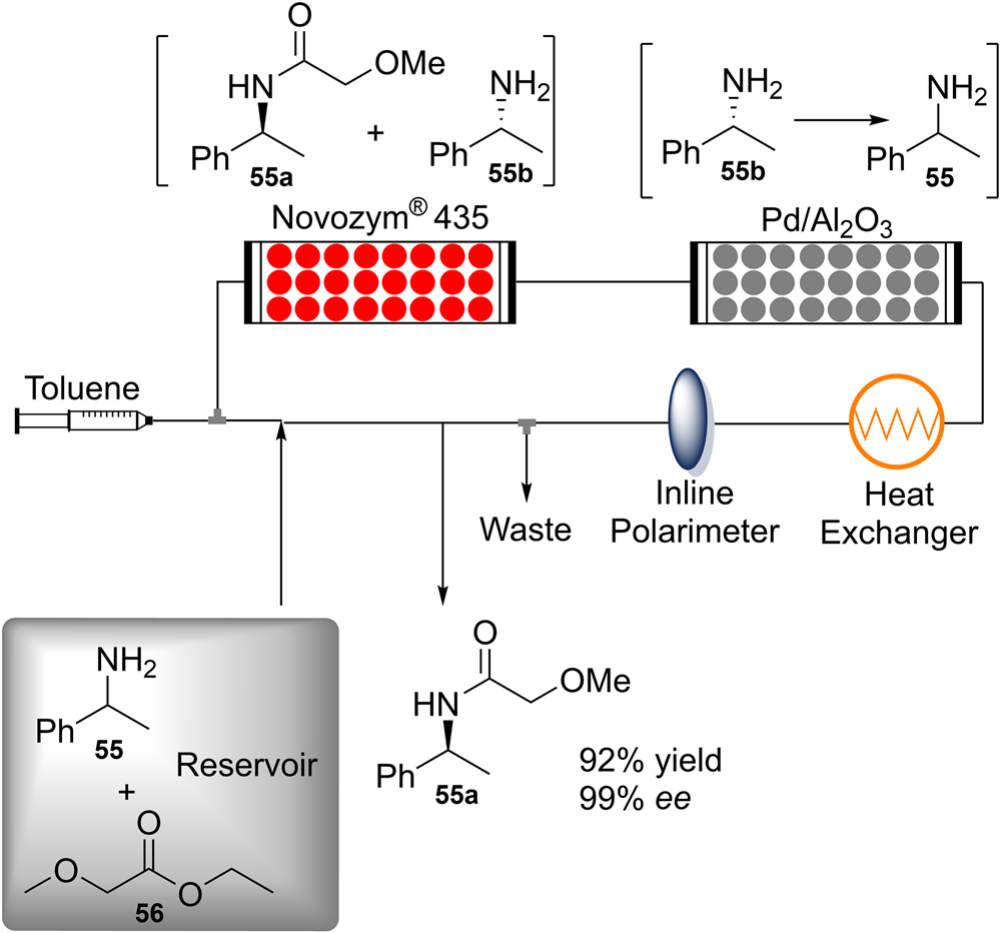
Schematic of the chemo‐enzymatic‐flash thermal racemization DKR platform.

More recently, Takle et al. published another example of a chemoenzymatic DKR, this time employing a flash thermal racemization protocol that enables the convenient racemization of primary amines (**55**) without requiring additional reagents (Scheme 16) [72]. The system, which is described as semi‐batch by the authors, is comprised of two main steps: first, the KR using the enzyme, and subsequently, the racemization using a metal catalyst. The process started with the pumping of the racemic amine from a reservoir alongside ethyl 2‐methoxyacetate (**56**) as the acylating agent and toluene as the solvent. The mixture, with a flow rate of 7 mL·min^−1^, was directed into the first PBR containing CalB immobilized on a resin (Novozym 435). There, the enzymatic resolution occurred at 40°C with a residence time of 35 s, yielding the acylated product (**55a**) while leaving behind the unreacted enantiomer (**55b**). After removing the ethanol byproduct with molecular sieves, as it interfered with the chemo‐catalyst, the mixture was directed toward the next PBR containing the Pd/Al_2_O_3_ dispersed in silicon carbide. In this column, the unreacted amine is racemized at 140°C with a residence time of 9 s. The low residence time of flow systems is crucial for this step due to the low stability of the imine intermediate. The outflow is then collected again in the reservoir, while the waste is removed with the help of a three‐way valve. Using this system, the researchers were able to convert 1 g of a racemic amine to the acylated product with 92% conversion and 99% enantiomeric excess. Although the authors observed constant enzyme activity over 1.5 h, the operational stability of the system over extended periods was not assessed.

**SCHEME 17:**
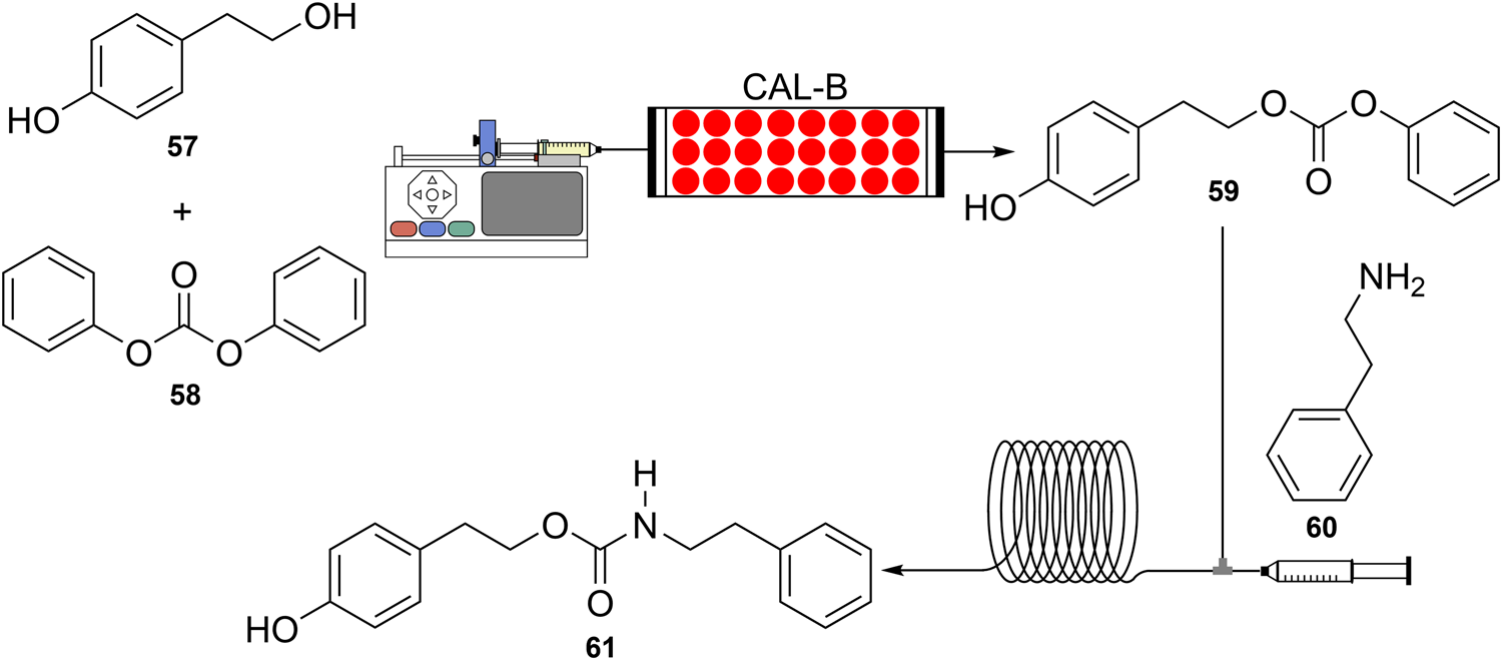
Setup of the telescoped chemo‐enzymatic flow process for the synthesis of carbamate **61**.

In 2024, Vicinanza et al. reported a chemo‐enzymatic flow synthesis of nature‐inspired phenolic carbonates and carbamates with applications as antiradical and antimicrobial agents (Scheme 17) [73]. These molecules possess antioxidant activity that is comparable to, or sometimes even greater than, that of the parent natural compounds. They can be applied as preservative agents in the pharmaceutical, food, and cosmetic industries. The reaction scheme consisted of two steps, starting with the enzymatic reaction to yield the carbonate, followed by heating in the presence of 2‐phenylethylamine to obtain the final corresponding carbamate. Tyrosol (**57**) was pumped alongside diphenyl carbonate (**58**), both dissolved in *tert*‐amyl alcohol at a flow rate of 33 µL·min^−1^, toward the first PBR. The column was packed with 450 mg of commercially available immobilized CalB. The temperature was set at 80°C, and the residence time of this step was 60 min. The result was the formation of the intermediate carbonate (**59**), which was mixed in a T junction with 2‐phenylethylamine (**60**), acting as the nucleophile, from another syringe pump at a flow rate of 33 µL·min^−1^, giving a total flow rate of 66 µL·min^−1^ before the mixture entered the next reactor. There, in a 2 mL coil reactor at 110°C with a residence time of 30 min, the reaction yielded the final carbamate (**61**). The whole system was pressurized at 20 psi, while the total residence time was 90 min. After the crude carbamate was formed and collected, the solvent was evaporated under pressure, and flash chromatography was performed to purify it, yielding the isolated product at a 45% yield. Utilizing a continuous flow chemo‐enzymatic system, the authors managed to selectively react the primary alcohol of the phenolic natural compound while preserving the integrity of the phenolic groups. Although the system demonstrated constant conversion over 8 h, the authors did not evaluate its stability over longer reaction times.

**SCHEME 18:**
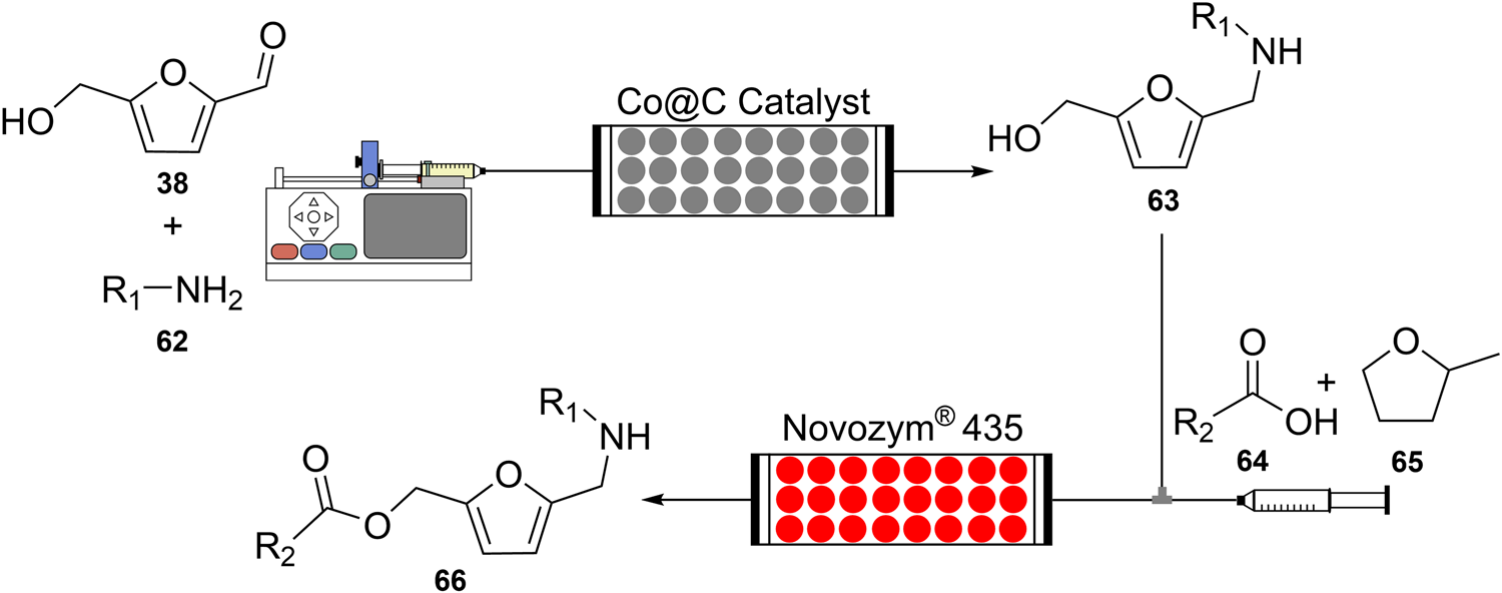
Continuous flow setup for the synthesis of amino‐esters **66** starting from HMF.

Another recent publication about chemo‐enzymatic synthesis in continuous flow was from Moriana Herraiz et al. on the production of amino‐esters, as precursors of ammonium salt‐based surfactants, from 5‐hydroxymethylfurfural (**38**) (HMF) (Scheme 18) [74]. This work presents a new strategy to valorize HMF into surfactant precursors by combining nonnoble metal catalysts with the highly selective enzymatic esterification of the hydroxymethyl group on the furan ring using aliphatic linear‐chain carboxylic acids. The process consists of two main steps: first, the reductive amination of HMF using a chemo‐catalyst, followed by the bio‐esterification of the amino alcohol in the presence of a lipase. Initially, a mixture of **38** and methylamine (**62**) in 2‐methyltetrahydrofuran (2‐MTHF, **65**) was directed toward the first fixed‐bed reactor containing the Co@C monodisperse nanoparticle catalyst. There, the reductive amination of HMF occurs through the activation of molecular hydrogen on the metal surface, facilitated by the carbon coating. The coating not only promotes the in situ reduction of surface oxide species but also prevents metal deactivation by protecting against overoxidation and agglomeration. The mechanism likely involves the formation of an imine intermediate between HMF and a primary amine, which is subsequently hydrogenated to yield the corresponding amine product (**63**). Using an HMF concentration of 3.5 wt%, the contact time was optimized to 8 h, providing excellent conversion and selectivity toward the corresponding amino‐alcohol. The reactor could run continuously for 95 h, performing exceptionally at an average yield of 95%. Then, for the second step, CalB immobilized on a resin (Novozym 435), along with molecular sieves, was placed in a flow reactor that was fed with a mixture of the amino‐alcohol and hexanoic acid (**64**) diluted with 2‐methyltetrahydrofuran (**65**). It is important to mention that the use of molecular sieves is necessary to eliminate any water that is released during the esterification, shifting the thermodynamic equilibrium toward the desired product. Again, the contact time was optimized to achieve yields of around 90%. This bio‐esterification step proceeded at 35°C with a flow rate of 1 mL·h^−1^. The continuous flow system was able to afford an overall yield of 85% of the desired amino‐ester derivative (**66**), which could be maintained for over 86 h of continuous operation.

**SCHEME 19:**
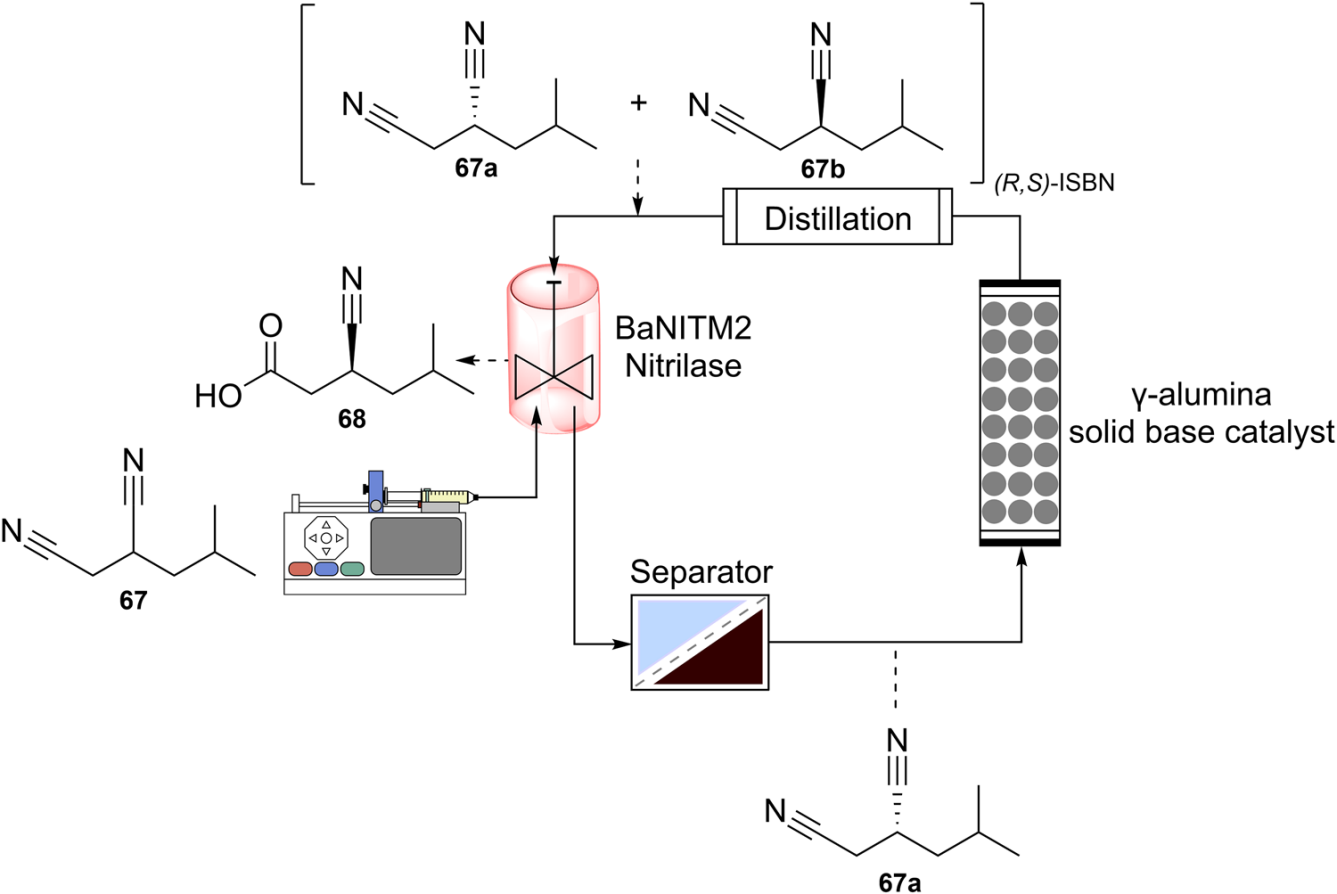
Kinetic resolution and racemization of isobutylsuccinonitrile (**67**) to yield (*S*)‐3‐cyano‐5‐methylhexanoic acid (**68**) as precursor of (*S*)‐pregabalin.

A chemo‐enzymatic process for the synthesis of the precursor of (*S*)‑pregabalin via nitrilase‐catalyzed hydrolysis combined with continuous flow racemization was published by Lin et al., (Scheme 19) [75]. Overall, this process is simple, robust, and achieves high space‐time yield, making it a promising candidate for potential industrial application. The process consists of two main steps. First, enzymatic enantioselective hydrolysis of (*S*)‐isobutylsuccinonitrile ((*S*)‐IBSN, **67b**) from its racemic mixture is performed in a continuous stirred‐tank reactor (CSTR) containing 100 g·L^−^^1^ of a crude preparation of the engineered nitrilase BaNITM2 immobilized on an epoxy resin. BaNITM2 exhibits high catalytic activity and excellent regio‐ and enantioselectivity toward isobutylsuccinonitrile, and was engineered by random mutagenesis and semi‐rational design. Under optimal conditions (pH 8, 35°C), the hydrolysis yields (*S*)‐3‐cyano‐5‐methylhexanoic acid ((*S*)‐CHMA, **68**), the precursor of (*S*)‐pregabalin, with 45% conversion and 99.5% ee within 8 h. The unreacted (*R*)‐IBSN (**67a**), mixed with *tert*‐pentanol, was pumped into a packed‐bed reactor (PBR), i.e., a water‐jacketed glass column containing 50 g of γ‐alumina (ASBC) at 40°C. In this step, racemization converts the unreacted (*R*)‐IBSN to racemic IBSN (**67a** + **67b**), which is separated by one‐step distillation and redirected to the CSTR for further hydrolysis. The PBR maintained 86% of its initial activity over 20 days under optimal conditions, achieving a space‐time yield of ∼7 mol·L^−^^1^·day^−^^1^.

### 1.2 Oxidoreductases

Oxidoreductases are a class of enzymes that catalyze redox reactions. In this context, the subclasses of the dehydrogenases and monooxygenases often require nicotinamide adenine dinucleotides (NADH or NADPH) and/or flavin mono‐ or dinucleotides (FMN or FADH_2_) as cofactors to enable electron transfer from an ultimate hydride donor molecule to a substrate acceptor or vice versa.

**SCHEME 20:**
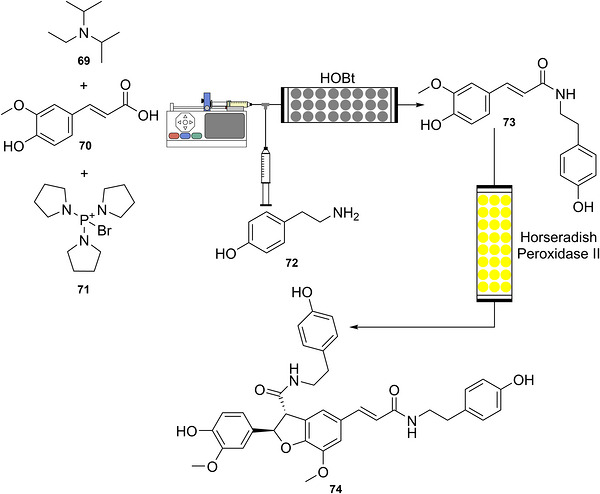
Schematic setup of the continuous flow synthesis of the Grossamide (**74**).

In 2005, Baxendale et al. reported one of the first examples of an oxidoreductase applied in a fully automated continuous‐flow reactor using a simple pumping system (Scheme 20) [76]. In their work, the group managed to prepare the natural product neolignan Grossamide (**74**) utilizing an immobilized horseradish peroxidase. They pumped ferulic acid (**70**) and bromo‐tris‐pyrrolidino‐phosphonium hexafluorophosphate (PyBroP, **71**) in the presence of N,N‐diisopropylethylamine (DIPEA, **69**), using tyramine (**72**) in THF as the coupling partner. The mixture passed through the first column, in which hydroxybenzotriazole (HOBt) was immobilized, to yield the intermediate amide adduct (**73**). An extra in‐line scavenging cartridge containing a sulfonic acid resin was placed after the first column to capture the unreacted amine. This step increased the purity of the amide product and provided an easy way of recovering the substrate. By eluting the cartridge with an excess of triethylamine in THF, the researchers were able to recycle the unreacted starting material. After the amide was obtained, it was diluted with a solution containing the hydrogen peroxide urea complex and sodium dihydrogen phosphate buffer (pH 4.5) in acetone–water (1:4, v v^−1^). Finally, the solution was passed through a third column packed with silica‐immobilized horseradish peroxidase (type II), which facilitated oxidative dimerization and intramolecular cyclization, yielding the final product. As a proof of concept, the fully automated synthesis of the neolignan natural product Grossamide (**74**) was accomplished using immobilized, packed‐column reagents and the oxidoreductase enzyme, thereby demonstrating the feasibility of integrating biocatalytic redox transformations into multistep continuous‐flow platforms and laying the groundwork for more advanced chemoenzymatic flow systems.

**SCHEME 21:**
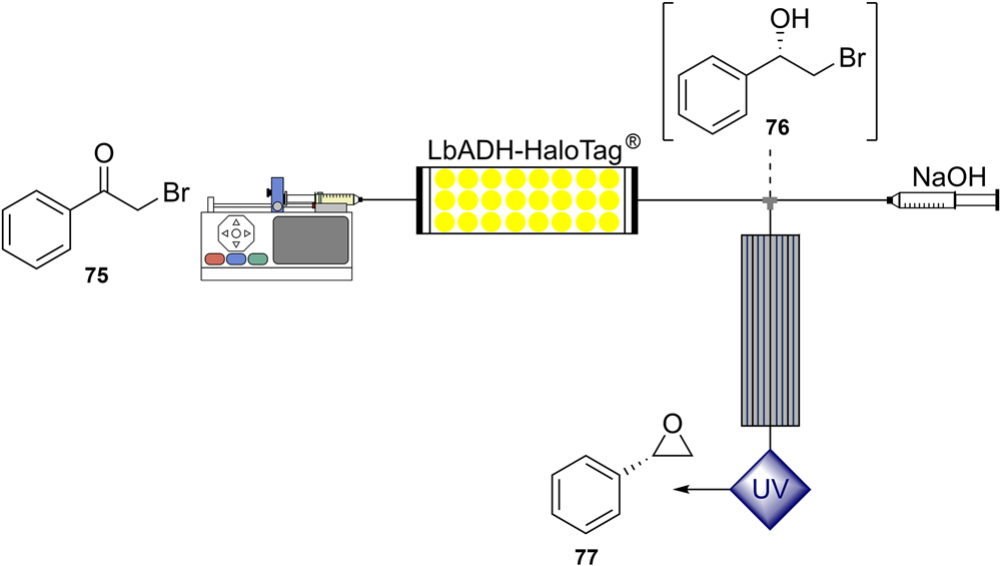
Asymmetric reduction of ketones by using immobilized HaloTag‐LbADH in flow.

More than a decade later, Döbber et al. reported the continuous biocatalytic production of chiral epoxides using an alcohol dehydrogenase from *Lactobacillus brevis* (Lb‐ADH) (Scheme 21) [77]. In this study, Lb‐ADH was genetically fused at its N‐terminus to HaloTag—a modified dehalogenase that enables rapid and highly selective covalent capture from crude cell extracts—affording a HaloTag–Lb‐ADH construct. This design enabled site‐specific and stable immobilization of the enzyme onto a functionalized resin without the need for extensive purification, thereby streamlining catalyst preparation for continuous‐flow application. The entire process is a two‐step chemo‐enzymatic synthesis that combines the bio‐reduction of 2‐bromoacetophenone (**75**) as the substrate to the corresponding chiral halohydrin with a cyclization in a second reactor to form a chiral epoxide. The process starts with the pumping of the substrate in THF, alongside NADPH, toward the PBR containing the biocatalyst at a flow rate of 30 µL·min^−1^ and a residence time of 19 min. After the first step, where the chiral halohydrin (**76**) is produced, an aqueous solution of NaOH is added to the stream at a flow rate of 30 µL·min^−1^. The mixture is then directed into a 1 mL glass microreactor where the halohydrin finally undergoes intramolecular cyclization under basic conditions (pH = 13) to form the final product. The produced (*S*)‐2‐phenyloxirane (**77**) can be continuously collected from the outflow in 98% yield and 98% enantiomeric excess. The continuous‐flow configuration provided a constant and well‐controlled product stream, underscoring the scalability and industrial relevance of the approach. Importantly, the immobilized enzyme maintained stable catalytic performance over four consecutive 300 min cycles before a gradual decline in activity became apparent.

**SCHEME 22:**
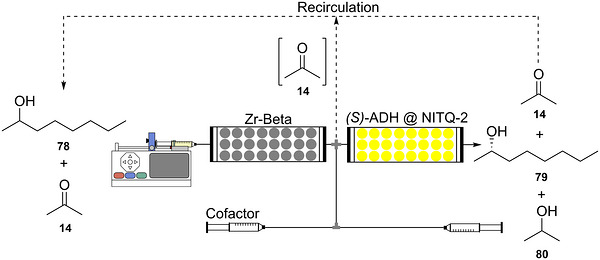
Oxidation–reduction system for the deracemization of alcohols.

In 2020, Carceller et al. published an article focused on the deracemization of racemic mixtures of alcohols by integrated heterogeneous chemo‐enzymatic catalysis in a fixed bed continuous operation (Scheme 22) [78]. The process consisted of two main steps. In the first one, a catalytic bed packed with 304 mg of dry Zr‐Beta catalyst on silica was used. The reactor was fed with a solution of *rac*‐2‐octanol (**78**) in acetone (**14**) to oxidize the former to the corresponding ketone, with a contact time of 25 h at a flow rate of 0.5 mL·h^−1^, while maintaining the temperature at 50°C. After exiting the first reactor, the solution of the resulting 2‐octanone was diluted with a mix of isopropanol (**80**) and phosphate buffer until a volume of 62 mL was obtained. At this point, the addition of the cofactor (NAD^+^) took place, and the solution was used to feed the second reactor. There, a purified alcohol dehydrogenase (Prelog ADH030 (*S*) or antiPrelog ADH270 (*R*)) was placed on a pure silica 2D zeolite that was functionalized with amino groups (NITQ‐2) to achieve immobilization through electrostatic interactions. This catalytic bed was fed with the mixture at the same flow rate with a contact time of 4 h at 25°C. Under these conditions, the enantioselective reduction of 2‐octanone led to 70% conversion due to the presence of acetone in the feed which can get reduced and competes with the desired reduction. When the researchers reduced or even completely removed acetone from the feed after the first step, the conversion increased to 96% with a 99% enantiomeric excess to (*S*)‐2‐octanol (**79**) (in the case of Prelog ADH030 (*S*)). The system operated for at least 40 h without any measurable loss of activity. Moreover, the oxidative–reductive deracemization proved applicable to various structurally diverse racemic alcohols (2‐dodecanol, 1‐cyclohexylethanol, etc.). Notably, the low boiling point of acetone facilitated its removal by flash evaporation, thereby improving conversion and enabling its reuse in the initial step. In summary, two fixed‐bed continuous reactors were designed and combined for deracemization of alcohols with yields above 95% and complete selectivity toward the desired *R* or *S* configured alcohols. Additionally, the system operated continuously for up to 16 days without detectable deactivation, highlighting its robustness and strong potential for scale‐up.

Two years later, Schober et al. published a study on the combination of asymmetric organocatalysis and biocatalysis in continuous flow, employing both sequential (Scheme 23a) and tandem modes (Scheme 23b) [79]. This work represents a highly promising platform for the synthesis of chiral 1,3‐diols and highlights the potential to extend this chemoenzymatic approach to additional transformations yielding otherwise less accessible products through flow chemistry. The sequential scheme starts with the enantioselective aldol reaction of 3′‐chlorobenzaldehyde (**81**) in the presence of a proline‐derived peptide that acts as an organocatalyst. The substrate and the organocatalyst (2 mol% loading) were mixed under continuous flow with 1.5 h residence time in a solvent mixture of isopropanol:acetone:buffer (3:2:1, v·v^−1^·v^−1^) to yield the aldol product. Then, prior to the enzymatic transformation, the reaction mixture was diluted. A purified solution of the enzyme—either ADH030 (*S*‐selective) or ADH270 (*R*‐selective), depending on the desired stereoisomer product—was added along with the cofactor (NADH) and an in situ cofactor regeneration system. The cofactor regeneration system consisted of d‐glucose dehydrogenase (GDH) and d‐glucose in potassium phosphate buffer. The biocatalytic step followed, with a residence time of 1 h, while the temperature in both steps was maintained at 25°C. This process gave access to all four possible stereoisomers of 1,3‐diol (**82**), with conversions ranging from 33% to 76%, good to high diastereomeric ratios, and an enantiomeric excess of >99% in all cases. For the tandem mode, three syringes containing the substrate, the organocatalyst (*S*,*S*), the enzyme (ADH030), and isopropanol as a sacrificial cosubstrate for NADH regeneration were directed into a coil reactor, where the combined organocatalytic aldol reaction and biocatalytic reduction took place. The reaction proceeded in a buffer aqueous system containing isopropanol and acetone as co‐solvents and had a residence time of 2 h at 25°C leading to the formation of the (1*R*,3*S*) product (**82a**) at 51% conversion, a d.r. of 8:1, and >99% ee for the desired 1,3‐diol. The authors performed an illustrative comparison between the two systems showcasing the strengths and weaknesses of each one as well as the particularities that need to be taken into consideration when incorporating the two different steps in cascade or in tandem. Differences such as the substrate, acetone, and isopropanol concentrations in addition to the cofactor regeneration system causing side reactions are aspects that need to be carefully thought out and executed in order for the process to be optimized, especially in the tandem mode. However, in neither case did the authors report data regarding the operational stability of the reactor system.

**SCHEME 23a:**
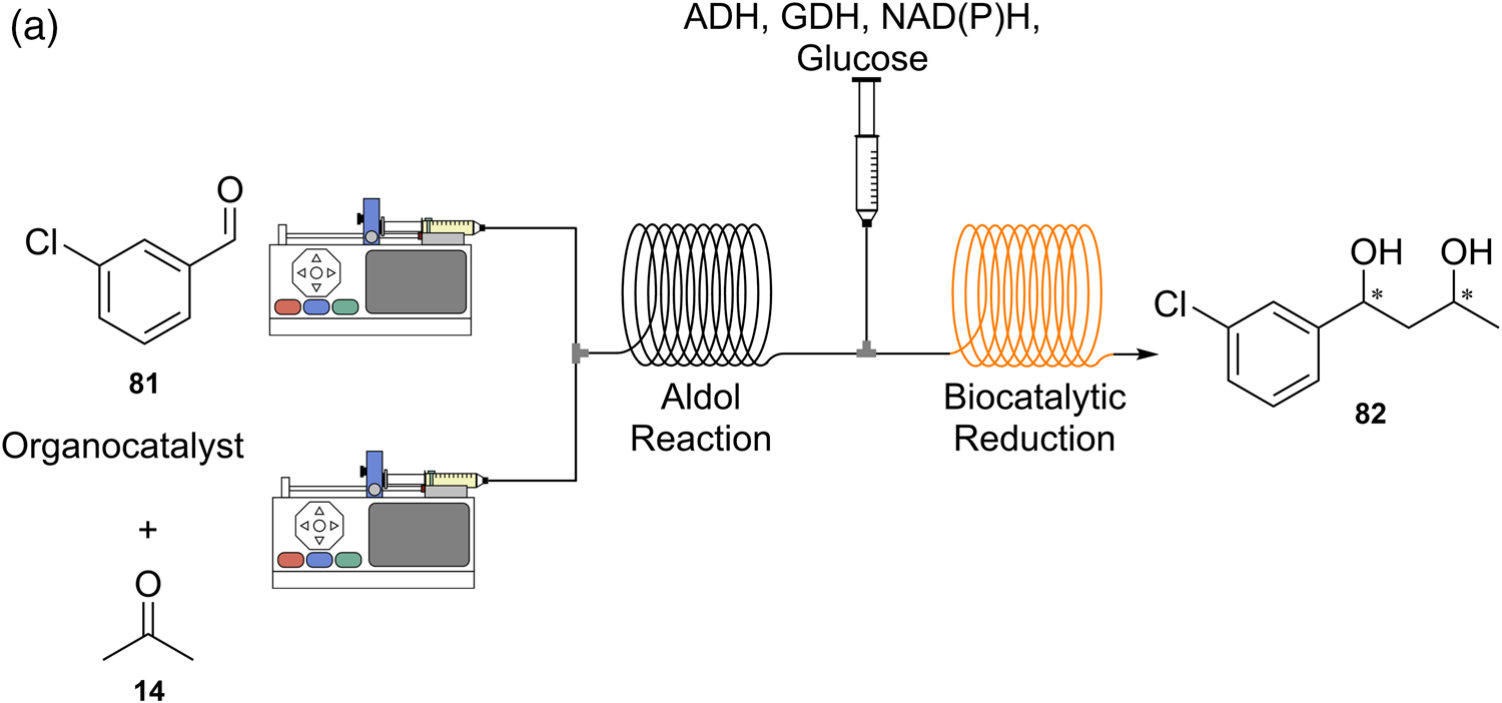
Schematic of the continuous‐flow setup for the sequential organocatalytic aldol reaction followed by ADH‐catalyzed reduction, including NAD(P)H regeneration via d‐glucose/GDH.

**SCHEME 23b:**
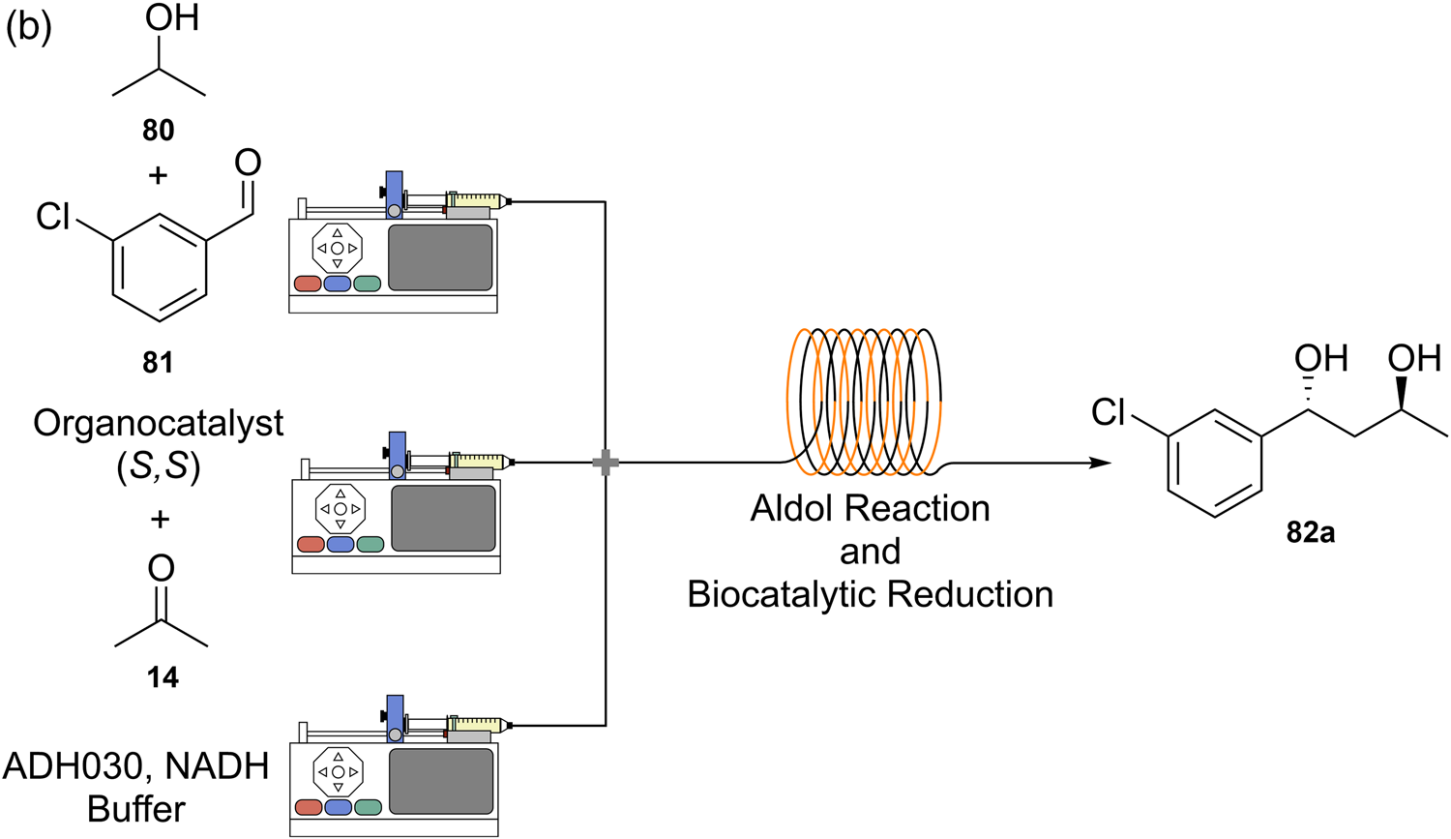
Flow setup for the tandem aldol‐ADH cascade setup with isopropanol‐based cofactor regeneration.

**SCHEME 24:**
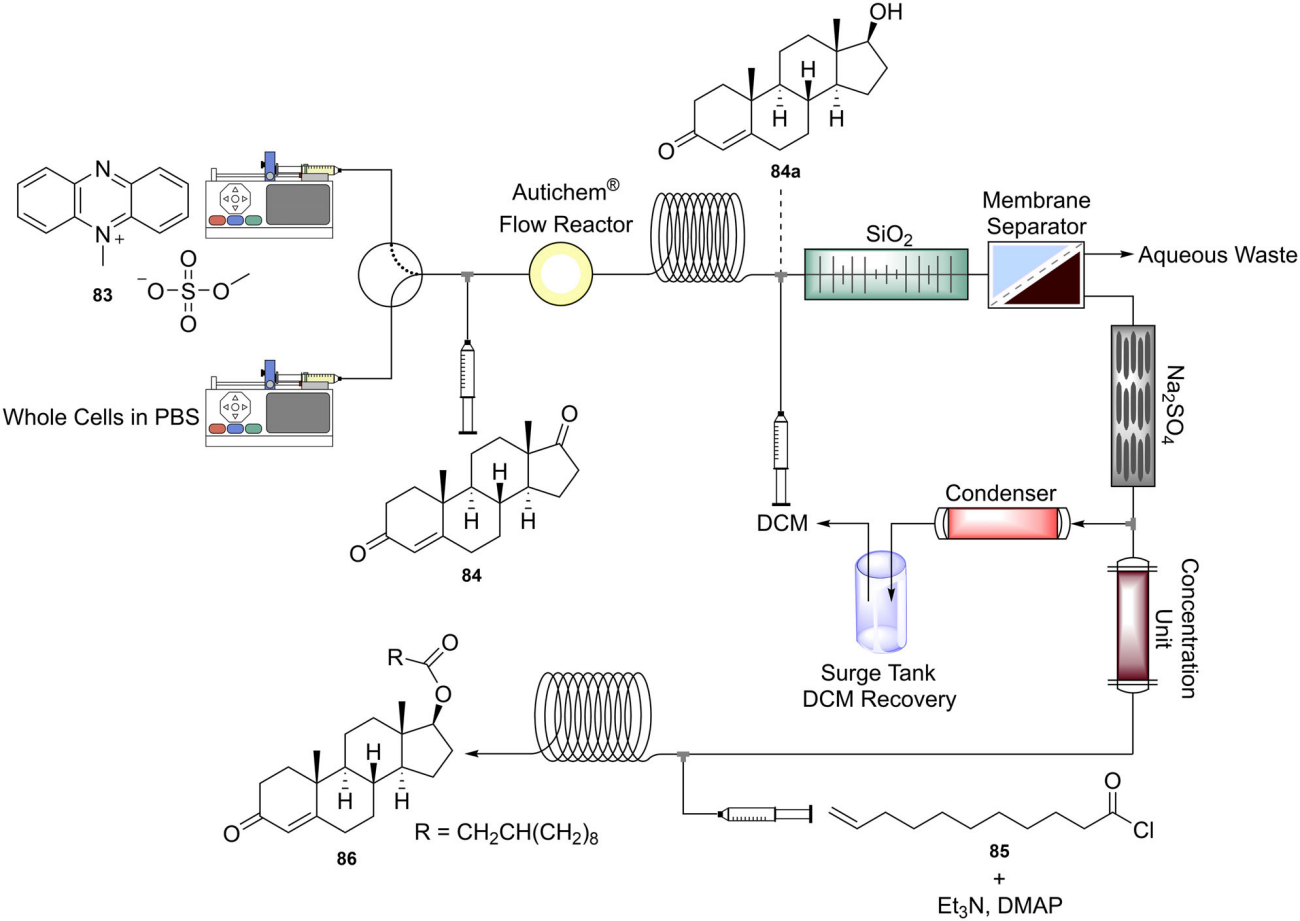
Setup for the continuous flow synthesis of (+)‐boldenone undecylenate (**86**).

In 2023, Zhang et al. published an article on the continuous‐flow asymmetric synthesis of anabolic‐androgenic steroids via a single‐cell biocatalytic cascade comprising Δ1‐dehydrogenation and C17β‐carbonyl reduction (Scheme 24) [80]. This work demonstrates an efficient telescoped synthesis of (+)‐boldenone undecylenate (**86**) in flow. The process begins with the enzymatic step, using whole *E. coli* cells as the biocatalyst. These cells co‐express a custom Δ1‐KstD (ketosteroid dehydrogenase) variant, ReM2, and a newly mined NADH‐dependent carbonyl reductase, 17β‐CR. Phenazine methosulfate (PMS, **83**)—an inducer of active oxygen formation and a stable electron‐transfer mediator for NADH—was mixed with 100 mg·mL^−^^1^ of whole cells in PBS and the commercially available substrate 4‐androstene‐3,17‐dione (**84**), before being directed toward the reactors. First, an Autichem flow reactor was employed for 17.5 min at 30°C to avoid clogging due to the precipitation of the poorly water‐soluble steroid compounds. Then, the mixture was directed into a PTFE coil reactor for another 12.5 min at the same temperature to yield the intermediate (+)‐boldenone (**84a**). By combining the efficient mixing of the Autichem reactor with the excellent mass‐transfer properties of the coil reactor, the researchers obtained (+)‐boldenone in 93.9% isolated yield and excellent selectivity (>99% de) using 20% DMSO, with the system operating continuously for 5 h. Notably, the space‐time yield of the enzymatic step in flow, at the same relative enzyme concentration as in batch, was an order of magnitude higher, reaching a STY of almost 11 g·L^−1^·h^−1^. This improvement was attributed to an enhanced mixing under continuous flow conditions and to switching from DMSO to DCM, which improved substrate and product solubility. The mixture was then diluted with DCM and passed through an integrated platform, which consisted of an online scavenger column to remove any cells and PMS, a liquid‐liquid membrane separator, a drying unit, and the solvent concentrator. These steps ensured a correctly concentrated DCM solution of (+)‐boldenone that was then headed toward esterification, avoiding any further purification. The effluent was then mixed with Et_3_N, DMAP, and 10‐undecenoyl chloride (**85**) before entering the PTFE coil reactor with a residence time of 25 min at 60°C to afford the final product ((+)‐boldenone undecylenate) in a 75% overall isolated yield.

**SCHEME 25:**
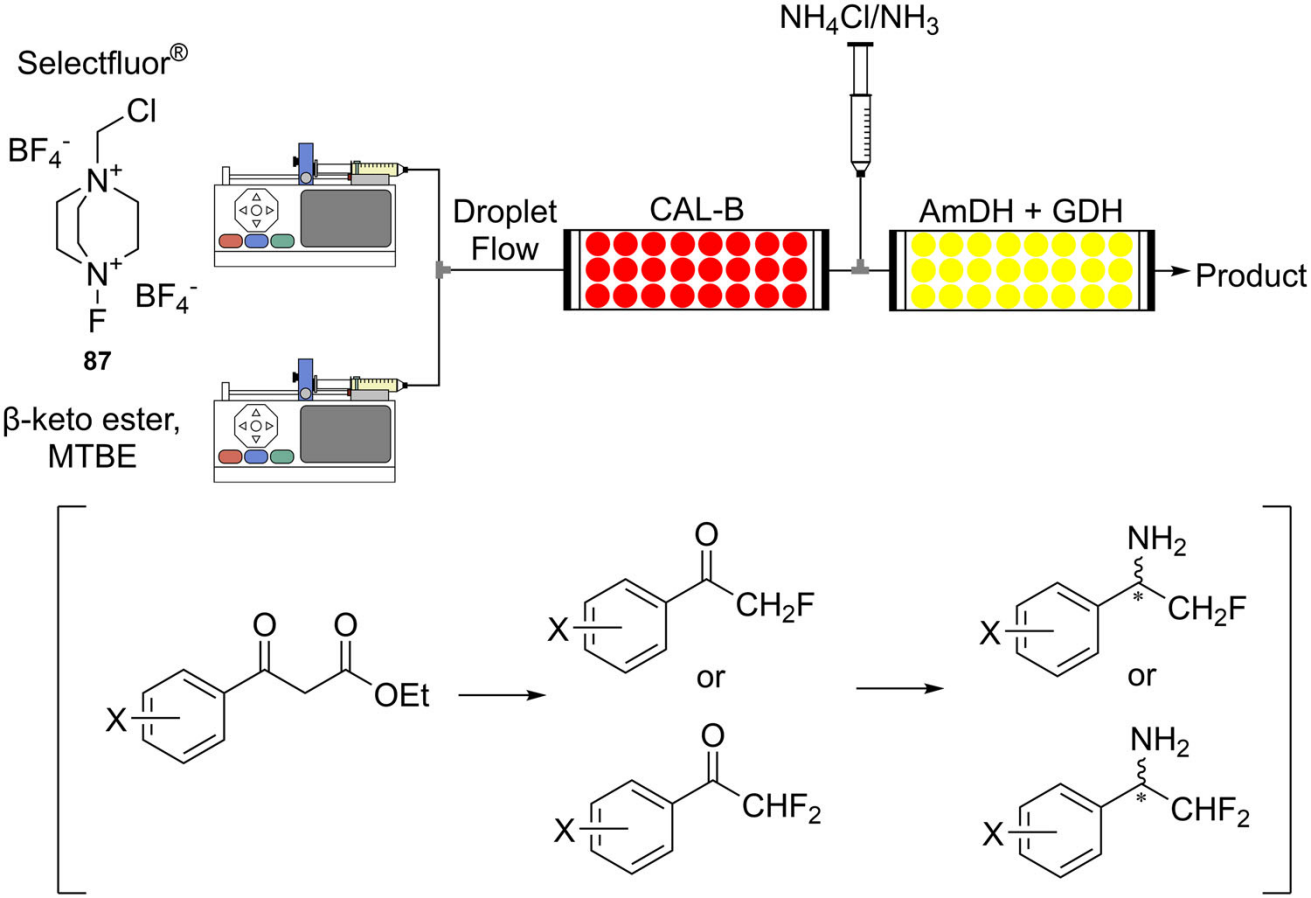
Chemo‐enzymatic enantioselective synthesis of chiral α‐mono‐ and di‐fluoromethyl amines in continuous flow.

Huang et al. reported another example showcasing the use of an oxidoreductase in the continuous‐flow chemo‐enzymatic enantioselective synthesis of chiral α‑mono‐ and di‐fluoromethyl amines (Scheme 25) [81]. The process consists of two main steps: a chemo‐enzymatic decarboxylative fluorination followed by an enzymatic reductive amination. Utilizing two pumps, the first one delivers the aqueous phase containing Selectfluor (a reagent used as a fluorine donor) and the second one is used for pumping the substrate (β‐keto esters) in the organic phase (MTBE). Taking advantage of a droplet flow, the mixture is directed into the first PBR containing the purified *Candida antarctica* lipase B (CalB) immobilized on magnetic organosilica nanoflowers, having a dendritic organosilica shell and a Fe_3_O_4_ core. In this PBR, the substrate undergoes decarboxylation and subsequent fluorination. Next, the reaction mixture is mixed with a NH_4_Cl/NH_3_ buffer solution before entering the next PBR. This second reactor contains co‐immobilized amine dehydrogenase from *Jeotgalicoccus aerolatus* (Ja‐AmDH, 197 mg_protein_·g^−1^_support_), with high catalytic activity and substrate specificity toward alkyl aryl ketones, and glucose dehydrogenase (GDH) for the regeneration of NADH cofactor. The two enzymes were immobilized on glutaraldehyde‐activated dendritic organosilica nanoparticles in an initial AmDH to GDH enzyme catalytic ratio (U U^−1^) of 5:1 (with an initial enzyme concentration of 1500 µg·mL^−1^). However, after co‐immobilization, the relative activities were found to be 1:1.8, which was similar to the optimized result of the same reaction in the free‐enzyme system (1:2). In the second reactor, the fluorinated alkyl aryl ketones were transformed in the corresponding α‑mono‐ and di‐fluoromethyl amines as the final product. The system used a flow rate ratio of the substrate pump to the Selectfluor pump of 10:1 at a flow rate of 0.1 mL·min^−1^ and a PBR containing a 1:1 (w/w) mixture of CalB@nanoflowers and nanoflower support. The obtained yield was 79%, a result attributed to the enhanced phase mixing. For the bienzymatic module, the fluorinated ketone and the NAD^+^ cofactor must diffuse from the oil and buffer phases, respectively, into the pores of the catalyst carrier and then into the enzymes’ active sites for the reaction to occur. Overall, in this water−oil−solid multiphasic chemo‐enzymatic process in continuous‐flow, almost quantitative conversion (at 100 mM substrate concentration) was achieved with a space‐time yield of up to 20 g L^−1^ h^−1^. This value was 35 times higher than the batch process using free enzymes. Additionally, 87% of the initial activity was retained after 96 h with a half‐life of 444 h, thereby demonstrating high operational stability.

Another application of an oxidoreductase in chemo‐enzymatic synthesis was reported by De Vitis et al. in 2017 for the synthesis of Captopril (Scheme 26a and 26b) [82]. Although this process is comprised of four steps, the combination of the enzymatic step with the chemical ones was not achieved. Nevertheless, their work is worth mentioning, not only because it aligns well with the broader context but also due to the innovative method the authors used to supply oxygen for cofactor recycling. The process starts with the biocatalyzed oxidation of a commercially available diol (**88**) using dried alginate beads of *Acetobacter Aceti* (Scheme 26a). The biocatalyst, in the form of whole cells, was immobilized and packed into a glass column, with no observable release of catalyst in the outflow. The oxygen required for the regeneration of the resting state of the cofactor was ensured by incorporating it as the gas in the air‐liquid segmented flow stream, which was formed before the contact with the immobilized catalyst. The residence time was set to 10 min at 28°C, yielding the corresponding acid (**89**) with 95% conversion and excellent enantiomeric excess (96–97%). The system exhibited adequate stability, maintaining consistent conversion over 10 h of continuous operation. To facilitate isolation of the acid, a catch‐and‐release strategy was implemented using a resin‐packed column, which selectively retained the product while allowing unreacted material to pass through. In the subsequent step (Scheme 26b), the produced acid was mixed with SOCl_2_ in toluene and DMF in catalytic amounts and was directed in a coil reactor for 30 min at 110°C. The result was the chlorination of both hydroxyl moieties. Afterwards, the chlorinated product (**90**) was mixed with l‐proline (**91**) in water and NaOH and headed toward another coil reactor for 3 min at ambient temperature. After the addition of HCl and EtOAc (**36**) followed by a membrane separation, they achieved the coupling of the two under 200 psi pressure. The coupled product (**92**), in the organic phase, was poured into a feed reservoir which was then mixed with NaSH in water and directed into another coil reactor for 30 min at 125°C. Again, after the addition of HCl, and a membrane separation under the same 200 psi pressure, the organic phase contained the desired Captopril (**93**). The total flow rate in each case was 0.33 mL·min^−1^ affording the final product at 50% yield after crystallization. Interestingly, the activity of the packed biocatalyst was retained entirely for the first 10 h, then it started to decrease, leading to a drop of the conversion from 95% to 80%. In this work, the authors converted a prochiral diol into a chiral intermediate with high enantiomeric excess via biocatalytic oxidation, followed by transformation into the desired product. The process could be further optimized by implementing a telescoped system using organic acid complexing agents, thereby eliminating the catch‐and‐release strategy and enabling a fully continuous preparation of Captopril.

**SCHEME 26a:**
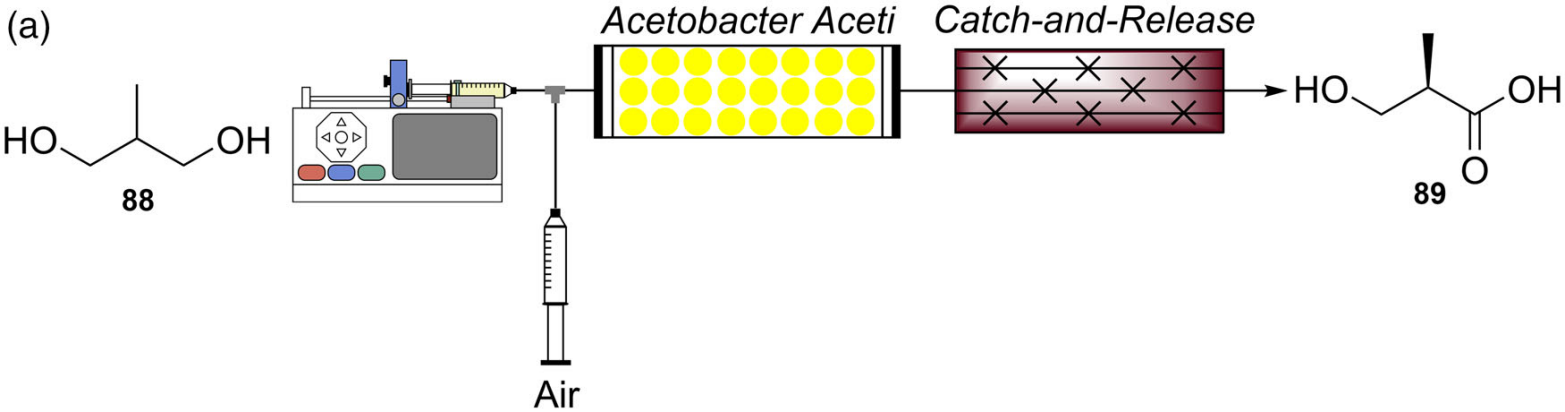
Enantioselective biocatalytic oxidation of 2‐methyl‐1,3‐propandiol and in‐line purification of the product.

**SCHEME 26b:**
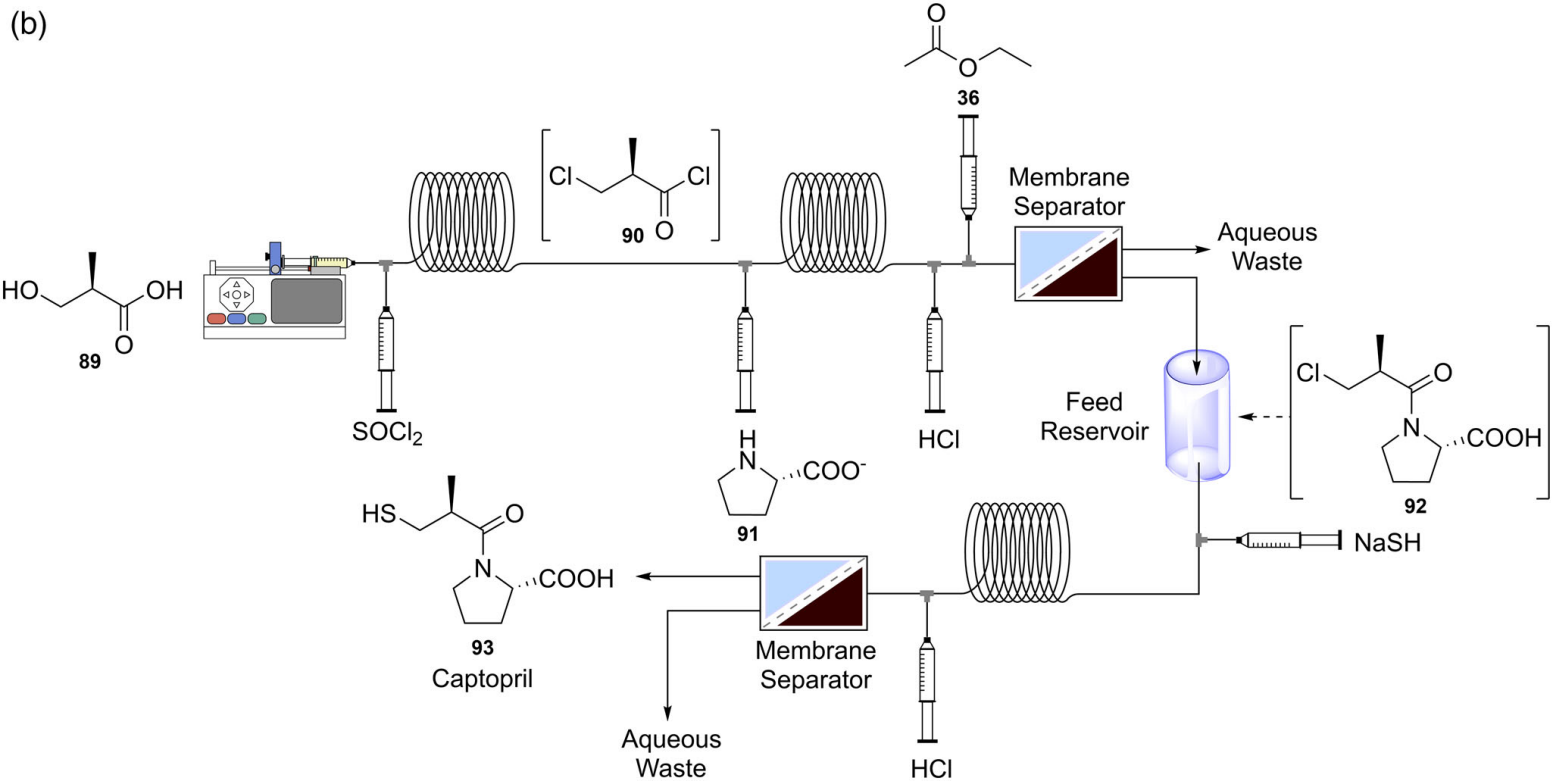
Flow setup for the three‐step chemical synthesis of Captopril (**93**).

### 1.3 Lyases

Lyases are enzymes that catalyze the breaking of chemical bonds, most often in the forward reaction, and their formation in the reverse reaction. This proceeds through an elimination reaction, which is not conducted via the pathways of hydrolysis and oxidation. A main difference between lyases and other enzymes is that they require only one substrate for the forward reaction (breaking of the bond) but two substrates to enable the reverse reaction. The type of lyases that are most commonly employed in continuous flow applications are decarboxylases and dehydratases.

**SCHEME 27:**
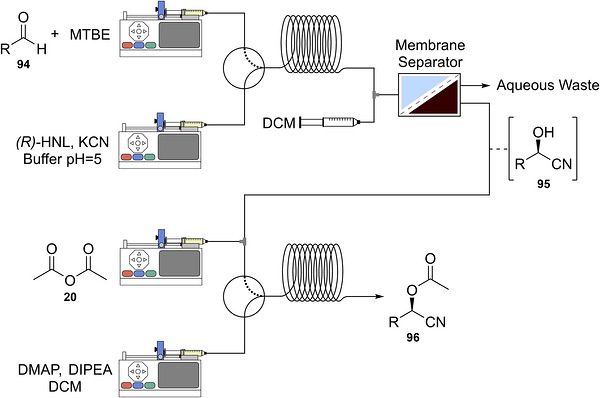
Schematic representation for the synthesis of protected mandelonitrile derivatives (**96**) in continuous‐flow.

One of the first appearances of a lyase in chemo‐enzymatic synthesis in flow was reported in 2014 by Delville et al. using an (*R*)‐selective hydroxynitrile‐lyase (Scheme 27) [83]. This work represents the first continuous‐flow cascade reaction to combine an aqueous chemo‐enzymatic step with an organic‐phase protection step, enabled by a membrane‐based phase separation module. In this system, the authors developed a two‐step cascade for the synthesis of protected mandelonitrile derivatives, seamlessly integrating biocatalysis with downstream chemical transformation in a continuous manner. The researchers used a 10% (v/v) crude cell lysate containing an *(R)*‐selective hydroxynitrile lyase (HNL) in the first step, followed by the addition of Ac_2_O (**20**) to achieve the final protected mandelonitrile derivative (**96**). Between the two steps, a PTFE‐based membrane separation took place to remove the aqueous phase. The base used for the protection step was added after Ac_2_O to prevent immediate racemization, and the reaction was quenched with water. The solvent system consisted of a biphasic mixture of methyl *tert*‐butyl ether (MTBE) containing the substrate (**94**) and a citrate buffer at pH 5 with KCN, to generate HCN in situ. For the second step, they added DCM to improve phase separation. In the process, the enzymatic reaction had a residence time of 12 min at 40°C, while the protection step had a residence time of 9 min at room temperature, resulting in an overall yield of approximately 60% and an enantiomeric excess of up to 98%. Finally, the authors decided to expand the scope of cyanohydrin (**95**) functionalization by trying different protecting groups. By replacing Ac_2_O with allyloxycarbonyl (Alloc), using the same scheme but omitting DMAP, they achieved a 62% yield with 87% ee of the Alloc protected mandelonitrile at 50°C. They also managed to successfully introduce the 2‐methoxyisopropyl (MIP)‐group in 68% yield and 97% ee. Notably, no information was provided concerning the operational stability of the enzyme under continuous‐flow conditions.

**SCHEME 28:**
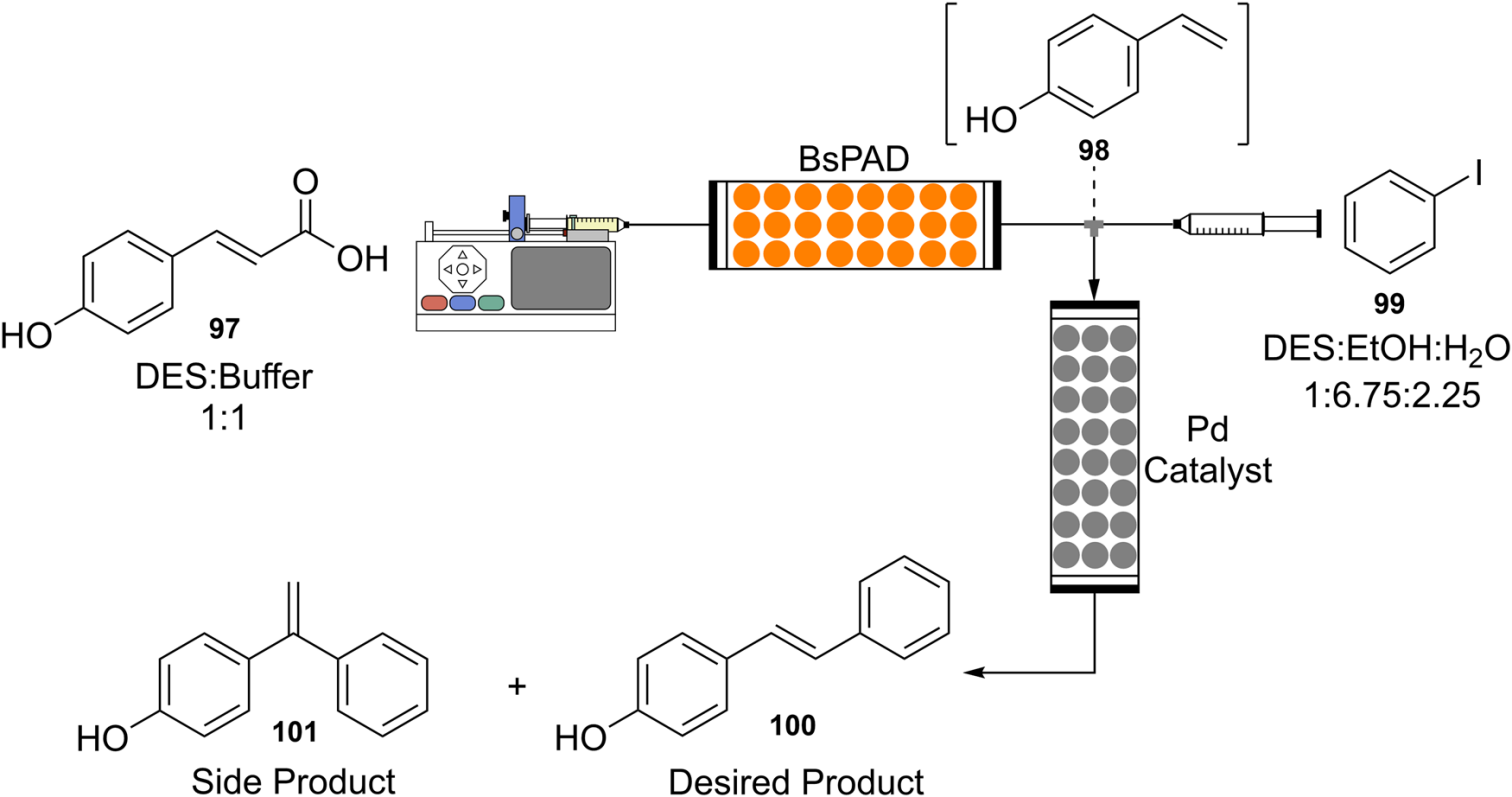
Flow scheme for the Bacillus subtilis (BsPAD)‐catalyzed decarboxylation and the subsequent Pd‐catalyzed cross‐coupling with aryl halide.

A few years later, Grabner et al. presented a chemo‐enzymatic tandem reaction in a mixture of deep eutectic solvent (DES) and water in continuous flow (Scheme 28) [84]. The process consisted of two steps, the first being the enzymatic decarboxylation of *p*‐coumaric acid (**97**) toward *p*‐hydroxystyrene (**98**) with a residence time of 30 min at 30°C, using 160 mg of phenolic acid decarboxylase from *Bacillus subtilis* (BsPAD) as a cell free extract immobilized in 2% alginate beads. The second step was a Heck reaction between the product of the decarboxylation and iodobenzene (**99**), carried out with a residence time of 45 min at 85°C, yielding the asymmetric (*E*)‐4‐hydroxy stilbene (**100**) in a 120 mm × 8 mm column packed with 6 g of a Pd catalyst. The relatively high catalyst loading required to achieve full conversion likely reflects the slow reaction kinetics. While complete conversion was maintained for 24 h, a thorough assessment of the long‐term stability of the Pd catalyst is necessary to determine the true sustainability of the process. The novelty of the process lies in the use of a DES, in which the immobilized BsPAD enzyme shows excellent activity. The system begins with a 1:1 mixture of DES and buffer to maintain pH 6, ensuring optimal enzymatic activity. For the Heck reaction, the final solvent composition is adjusted by adding ethanol, water, additional DES, and iodobenzene prior to the reaction. The flow rate was set to 45.5 µL·min^−^^1^ for each pump, resulting in a total flow rate of 90 µL·min^−^^1^. The entire system was maintained at 5 bar pressure to keep the produced CO_2_ dissolved, thereby preventing bubble formation. Thorough flushing was performed to remove any loosely bound enzyme and a filter was added to capture denatured fragments, thereby preventing clogging. Continuous operation was sustained for 16 h, resulting in a cumulative 20% yield of the final product. The yield was lower than that of the batch process due to the lack of isomerization of the side product (**101**) into the more stable desired product in the continuous flow setup. Nevertheless, the product could easily be isolated by evaporating ethanol, followed by extraction in MTBE, resulting in a space‐time‐yield of 5 g L^−1^ h^−1^ for decarboxylation and 0.5 g L^−1^ h^−1^ for Heck coupling.

In 2022, Gianolio et al. showed the application of a different decarboxylase, specifically a tyrosine decarboxylase from *Lactobacillus brevis* (Lb‐TDC) (Scheme 29a) [85]. The soluble expression of Lb‐TDC was successfully achieved and the enzyme was immobilized on a methacrylic support (ReliSorb 400(SS)), with the optimal conditions being 5 mg of purified enzyme per gram of support. The enzyme activity reached up to 33 U·g^−1^. It showcased excellent stability when stored at 4°C and successfully maintained its activity for over 56 reaction cycles (one cycle defined as the passage of one column volume). After 103 reaction cycles, the conversion was still above 95%, remaining above 90% after 8 h, which corresponded to 192 reaction cycles. The reaction scheme consisted of two steps. The first step was the enzymatic decarboxylation of l‐tyrosine (**102**) using a 5 mM l‐tyrosine disodium salt hydrate and 0.2 mM PLP (**103**) in 200 mM sodium acetate buffer at an inlet flow rate of 0.54 mL·min^−1^. The second step was a chemical reductive alkylation to obtain the final product, hordenine (**105**). For this step, the researchers tested two different approaches. In the first, a mixture of 62.5 mM formaldehyde and 2.5% v v^−1^ acetic acid in MeCN was used, followed by a solution of sodium triacetoxyborohydride (STAB, **104**) in MeCN. The reaction mixture was passed through a 10 mL coil reactor at 25°C. Finally, NaOH and EtOAc (**36**) were added to process the mixture and extract hordenine into the organic phase. Using 12.5 equivalents of formaldehyde and 12 equivalents of STAB in 75% v v^−1^ MeCN at room temperature yielded a 96% conversion with a residence time of 5 min. Notably, there was no requirement for a back pressure regulator or temperature control in the flow setup. The achieved space‐time‐yield was around 3 g_hordenine_ L^−1^·h^−1^, which is 50% higher than what had previously been reported. However, the presence of MeCN was unwanted due to the generation of toxic products (HCN and NaCN). Therefore, picoline borane (pic‐BH_3_) was tested as an alternative and “greener” reducing agent (Scheme 29b). A total of 300 mg of pic‐BH_3_ was packed with an equivalent amount of celite into a PBR used as a heterogeneous reducing module in flow. This time, a solution of 203 mM of formaldehyde in 250 mM sodium carbonate was added after the enzymatic step. The mixture was passed through the PBR containing the pic‐BH_3_, and the final mixture was treated only with EtOAc (**36**) to yield hordenine (**105**) in the organic phase. This system, with a residence time of 2.5 min, achieved a higher space‐time yield of 11 g_hordenine_·L^−1^·h^−1^, which is four times greater than the first system, while also utilizing a well‐established, nontoxic alternative. An important aspect of this work is the E‐factor of the system with pic‐BH_3_ suspended in celite, which is 36 and falls well within the ideal pharmaceutical industry standards (around 25–100) [86]. It is also worth noting that this process was profitable, resulting in a material value increase of around 200‐fold (from l‐tyrosine to hordenine).

**SCHEME 29a:**
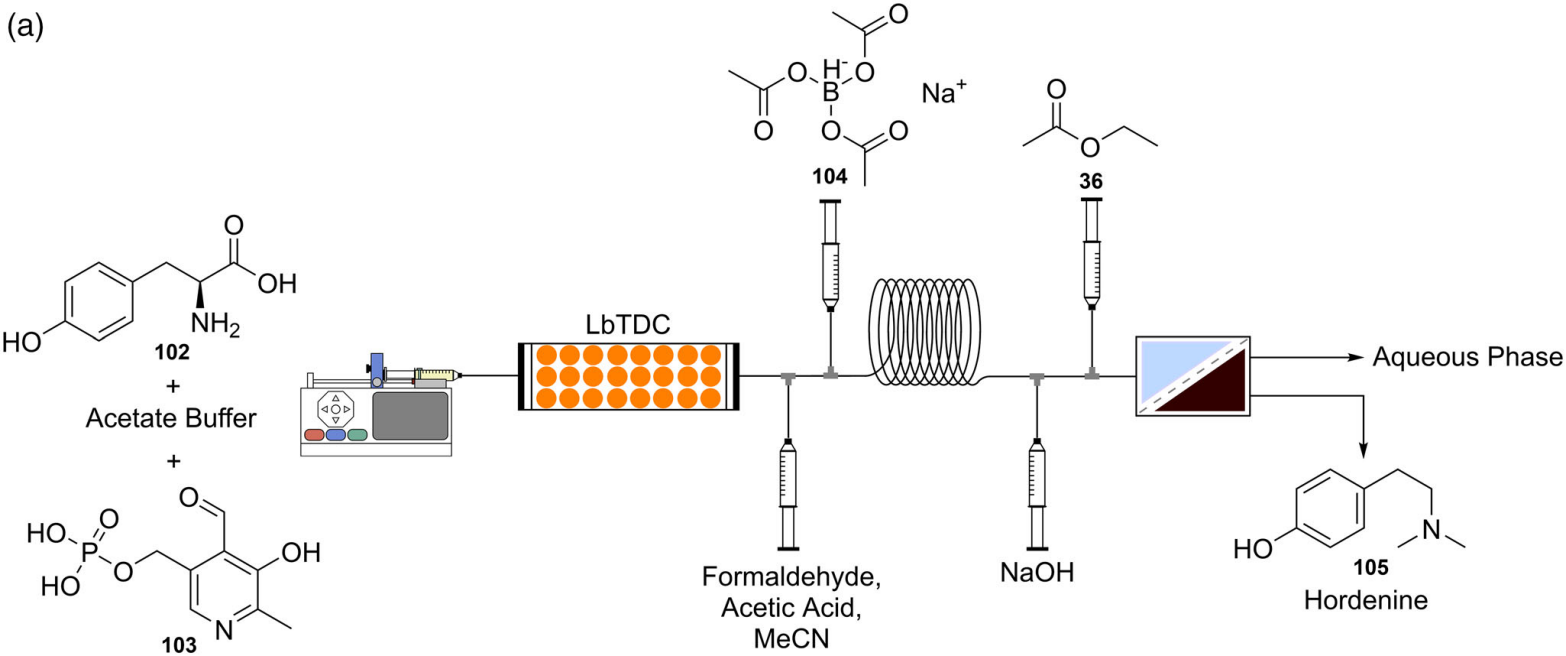
Scheme of the continuous flow chemo‐enzymatic system for hordenine (**105**) production using STAB.

**SCHEME 29b:**
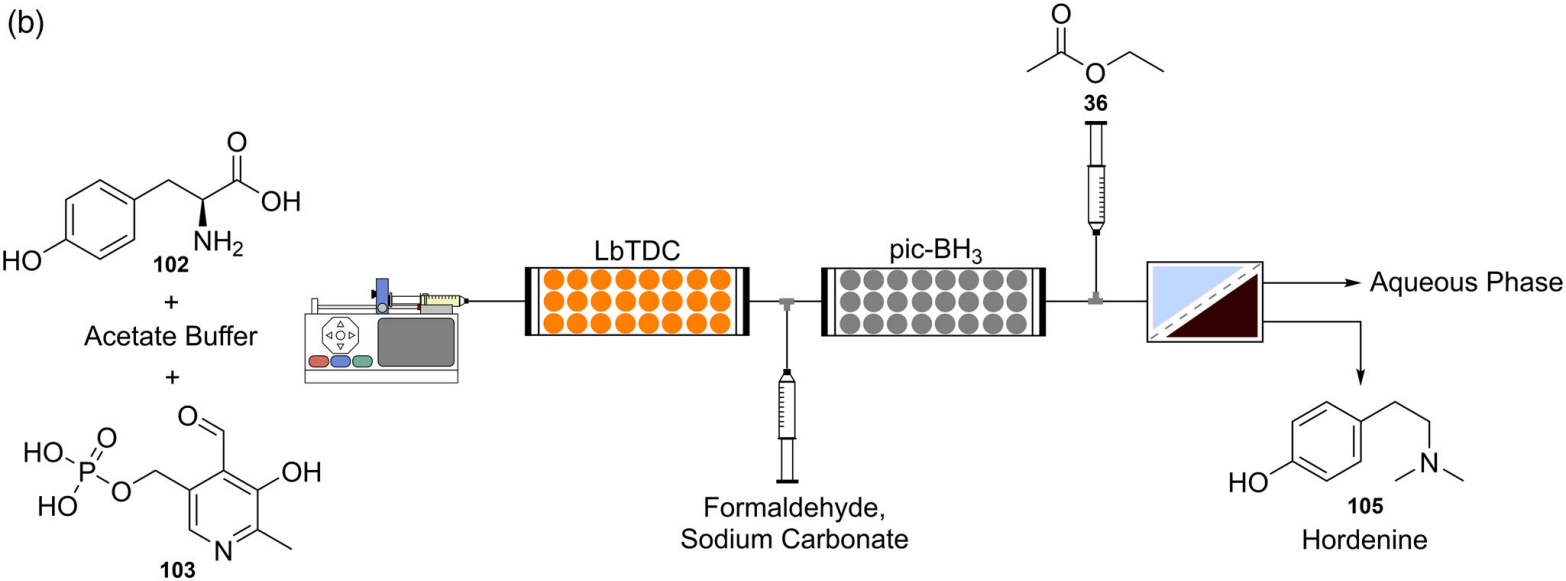
Scheme of the continuous flow chemo‐enzymatic system for hordenine (**105**) production using pic‐BH3.

**SCHEME 30:**
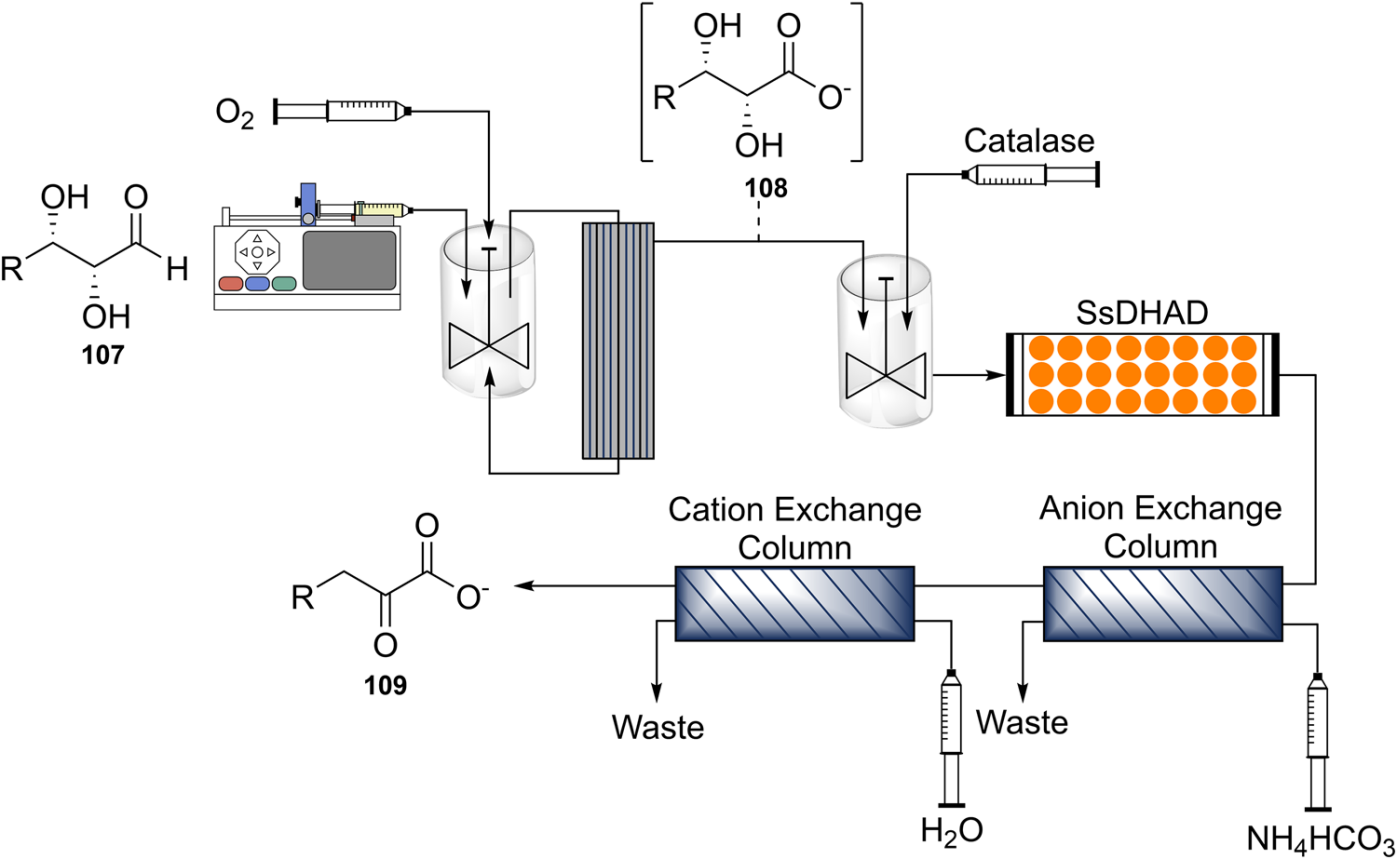
Flow setup for the chemo‐enzymatic synthesis of KDS.

Finally, Sperl et al. tested a different type of lyase: a dihydroxyacid dehydratase from *Sulfolobus solfataricus* (SsDHAD) (Scheme 30). Although the project initially aimed to develop a one‐pot, two‐step batch process, numerous incompatibility issues between the two catalysts prompted the researchers to explore a continuous‐flow approach. This shift highlights the advantages of chemo‐enzymatic continuous‐flow processes, particularly their ability to accommodate various combinations of heterogeneous and biocatalysts operating under distinct conditions. The final process was carried out as a fed‐batch continuous‐flow system in combination with catalase [87]. This relatively simple process consists of two main steps, integrating the intermediate removal of the undesired H_2_O_2_. The process begins with a stirred fed‐batch reactor containing an Au catalyst immobilized on alumina, where a gold‐catalyzed direct oxidation of the sugar acid (**107**) occurs at the expense of molecular oxygen; H_2_O_2_ is generated as co‐product on the Au surface. An automatic titrator is used to keep the pH at 9, which is optimal for the catalyst. Next, the reaction mixture is passed through a hollow fiber module and then into another stirred reactor containing catalase and an automatic titrator, ensuring optimal conditions for H_2_O_2_ removal. If this step is omitted, the enzyme risks denaturation. The reaction mixture then passed through a column reactor containing the immobilized biocatalyst for the enzymatic dehydration step. The enzyme is immobilized in purified form into Ni‐Sepharose column, kept at 50°C. After that, two rapid purification steps using an anion and a cation exchange column yield the pure product (**109**). At a flow rate of 1 mL·min^−1^ and using l‐arabinose as the starting material, pure l‐2‐keto‐3‐deoxyarabonate (L‐KDA) was obtained at 58% yield. For d‐glucose, at 0.3 mL·min^−1^ flow rate, a yield of 86% was achieved, demonstrating variations in productivity depending on the specific activities of the enzyme toward different substrates. As the authors mention, the promiscuous nature of the dehydratase, combined with the broad substrate range of the gold catalyst could enable the synthesis of even more 2‐keto‐3‐deoxy sugar acids (KDS) derivatives.

### 1.4 Transferases

Transferases catalyze the transfer of specific functional groups (e.g., methyl, glycosyl, acyl, alkyl, phosphate, sulfate, amino groups) from a donor molecule to an acceptor molecule [88, 89, 90, 91, 92, 93]. Transferases are essential for life, as they participate in hundreds of distinct biochemical pathways. In industry, the most widely used transferases are transaminases, which are employed for the synthesis of chiral amines, and glycosyltransferases, which are used for the synthesis of oligosaccharides—both classes of compounds being important for the pharmaceutical and fine chemical industries [88, 89, 90, 91].

To enhance the applicability of transferases in an industrial setting, chemo‐enzymatic reactions under continuous flow have been investigated.

**SCHEME 31:**
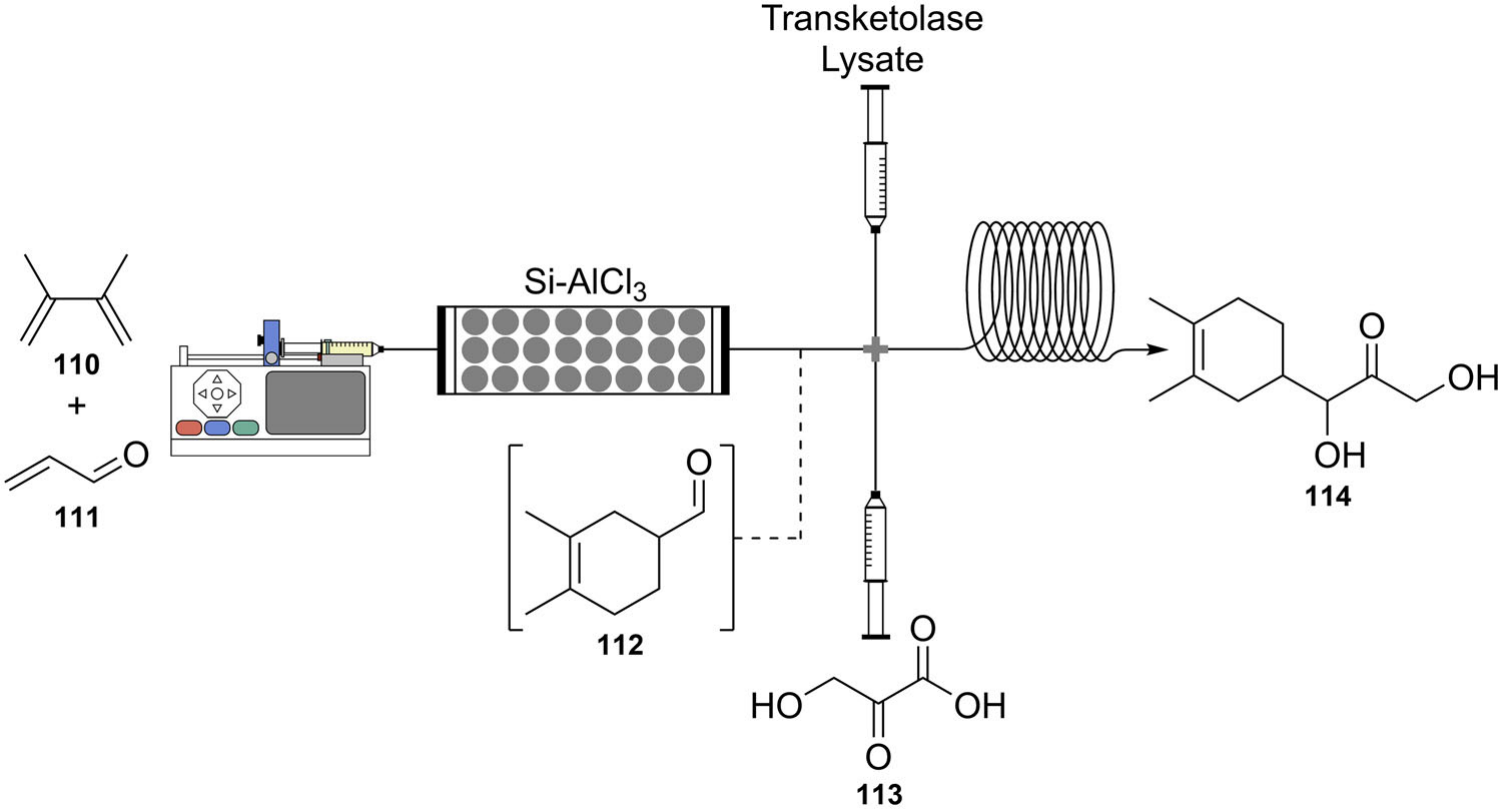
Setup for the synthesis of DCDHP (**114**) in the Diels–Alder–transketolase microfluidic cascade system.

For instance, Santos et al. reported the combination of a Diels–Alder reaction with a transketolase‐catalyzed step (Scheme 31) [94]. This setup highlights the advantages of continuous‐flow chemistry and microreactor technology for conducting chemo‐enzymatic cascade reactions, eliminating the need for protection steps and enabling the deconvolution of distinct operational windows to prevent enzyme inhibition. The process comprised two steps, beginning with a mixture of 2,3‐dimethyl‐1,3‐butadiene (**110**) and acrolein (**111**) passed through a packed‐bed reactor containing the Diels–Alder catalyst (Si–AlCl_3_). Subsequently, using a four‐way connector, the researchers fed the mixture with the transketolase and the hydroxy‐pyruvate, the latter of which acts as the co‐substrate. The enzyme was used in the form of a cell free extract that had been incubated for 30 min with the cofactor thiamine diphosphate (ThDP). Quantitative substrate conversion was achieved toward 1‐(3,4‐dimethylcyclohex‐3‐en‐1‐yl)‐1,3‐dihydroxypropan‐2‐one (**114**, DCDHP), producing a fivefold increase yield when compared to the batch process. Using a flow rate of 4 µL·min^−1^, the reaction was initiated by pumping an equimolar mixture of 100 mM of 2,3‐dimethyl 1,3‐butadiene (**110**) and acrolein (**111**) in acetonitrile through the Si‐AlCl_3_ PBR, yielding the Diels‐Alder product (**112**). The resulting outflow was then diluted in the four‐way connector with 5 mM of 3‐hydroxy‐2‐oxopropanoic acid (**113**, HPA) in 25 mM Tris‐HCl buffer (pH 7) at a flow rate of 4 µL·min^−1^, along with the transketolase lysate solution at 32 µL·min^−1^, summing up to a total flow rate of 40 µL·min^−1^ before entering the coil reactor. The outlet stream of the coil microreactor contained the DCDHP product (**114**), which was analyzed via HPLC for HPA depletion. No data on the selectivity or stability of the system were reported in the study.

**SCHEME 32:**
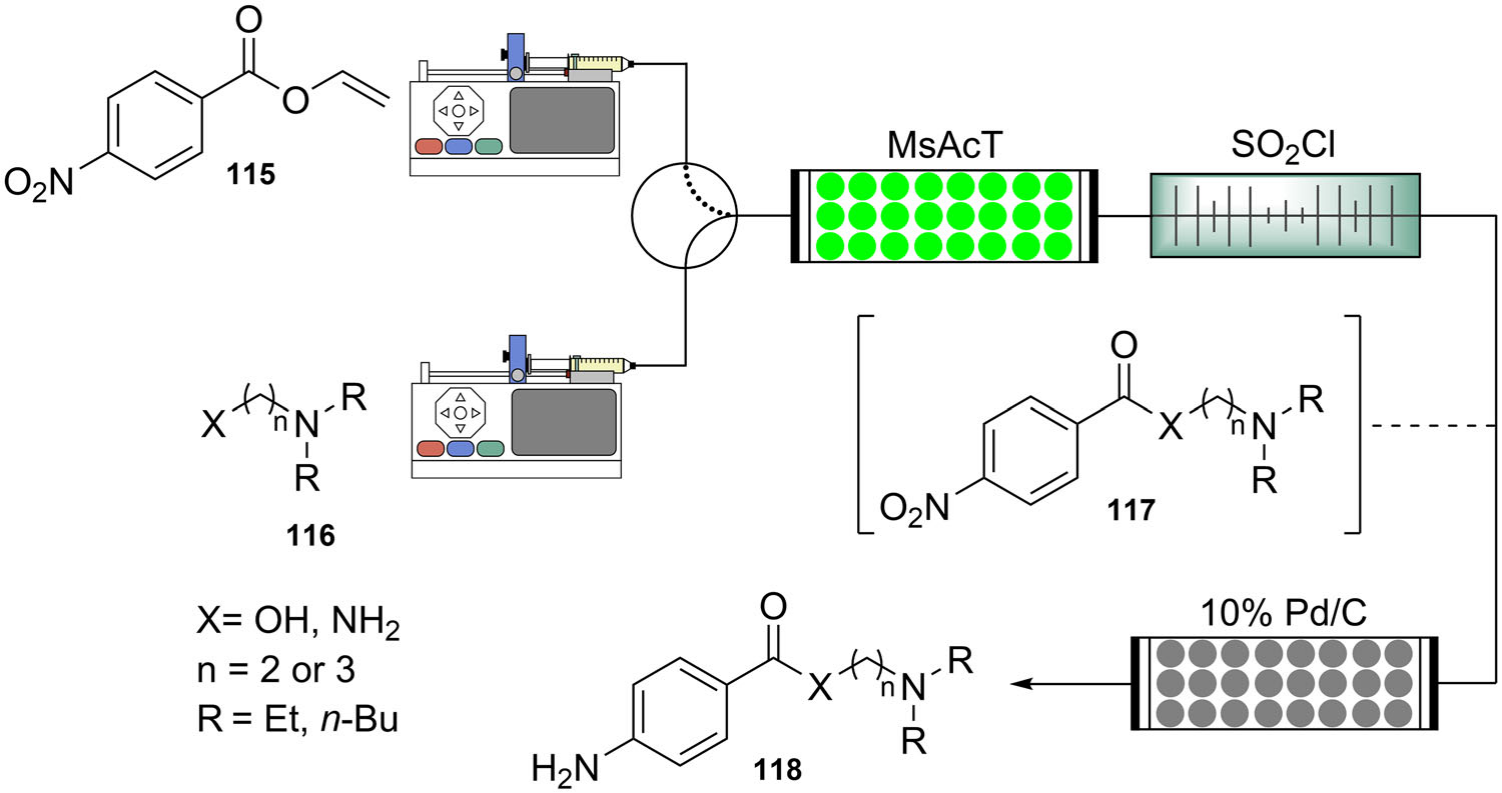
Flow setup for the chemo‐enzymatic synthesis of high value amides and esters.

In the same year, Annunziata et al. presented a chemo‐enzymatic flow synthesis of high‐value amides and esters using a versatile acyltransferase from *Mycobacterium smegmatis* (MsAcT), employed in a pure organic solvent (Scheme 32) [95]. This work demonstrated the enzyme's high stability and reusability. The researchers also highlighted the low cost of preparing certain APIs (procainamide, procaine, butacaine). The process is easy to handle, fast, safe, and robust, and by implementing larger, commercially available packed‐bed reactors (PBRs), it offers good scalability. The process consisted of two main steps integrating an in‐line purification between them. Starting with the enzymatic reaction, 2.5 g of purified MsAcT was immobilized on glyoxyl‐agarose in a 1 mg_enzyme_·g^−1^_support_ ratio. Using a T‐piece connector, 0.25 M vinyl 4‐nitrobenzoate (**115**) and 0.5 M N,N‐diethylethane‐1,2‐diamine (**116**)—both in toluene, to limit hydrolysis of the former and facilitate nucleophile access—were mixed and directed to the bioreactor. There, the reaction was completed within a residence time of 7 min at 28°C, with no formation of side products, affording the intermediate amide (**117**). The system achieved a productivity of 0.4 g·h^−1^·mg^−1^_enzyme_ and ran for 6 h, showing only a slight decrease in the conversion at the end of the experiment (from 97% to 95%). Subsequently, the in‐line purification step was carried out using polymer‐bound sulfonyl chloride to trap any excess nucleophile and enable product isolation by simple solvent evaporation, thereby increasing protocol automation and reducing work‐up time. Finally, a flow hydrogenation of the isolated and purified intermediate amides was performed using a 10% Pd/C cartridge to reduce the nitro group to an aniline in the final products (**118**). The reduction was conducted at a flow rate of 0.8 mL·min^−1^, 60°C, and 10 bar, giving excellent yields (>99%).

**SCHEME 33:**
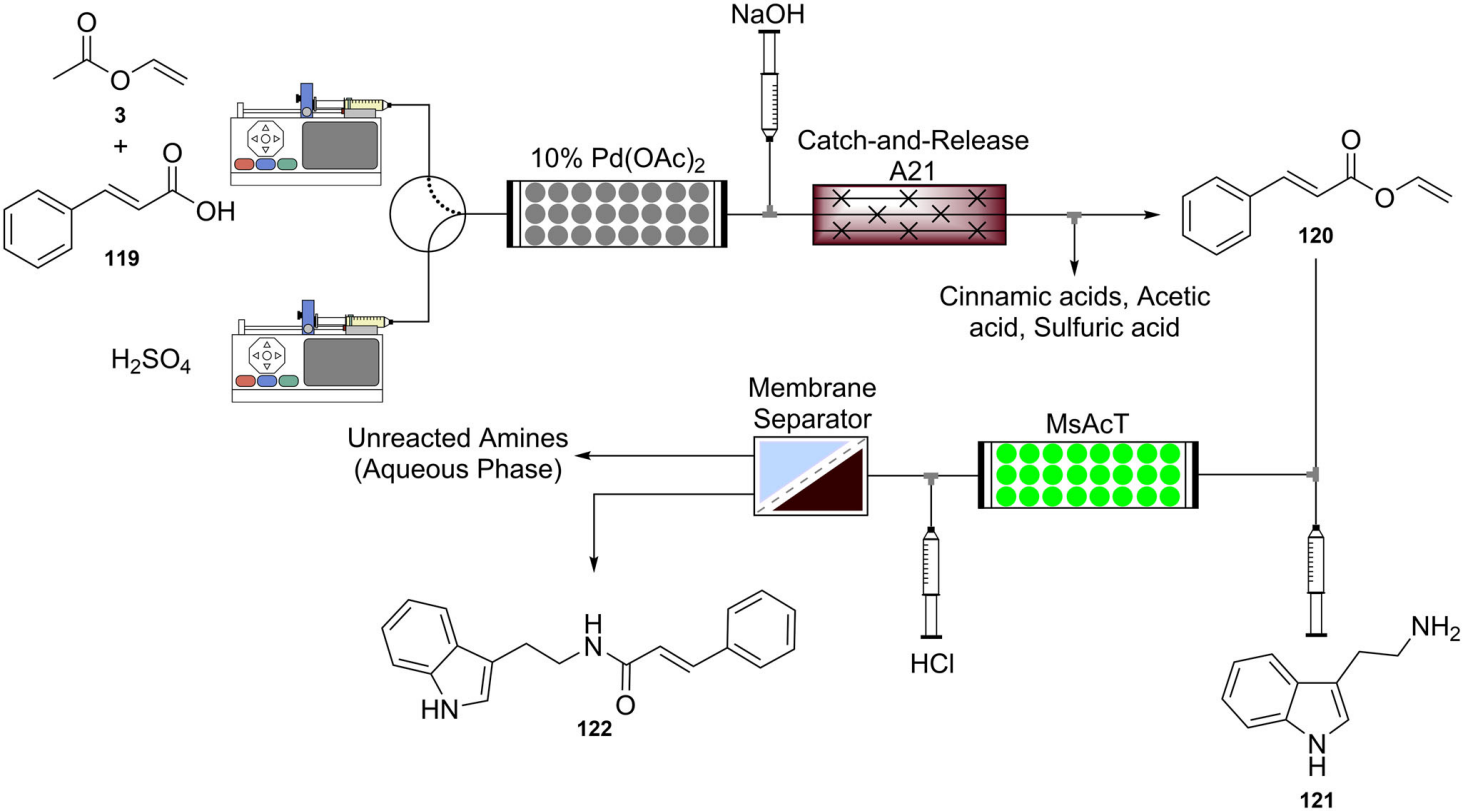
Flow setup for the preparation of cinnamoyl tryptamines (**122**).

In 2021, Padrosa and Contente introduced a two‐step chemo‐enzymatic continuous‐flow synthesis of cinnamoyl tryptamines, once again using the transferase MsAcT (Scheme 33) [96]. This study demonstrates that combining the advantages of flow chemistry with closed‐loop strategies enables the recovery of unreacted substrates while minimizing solvent consumption, thereby creating a significantly more sustainable process. The process started with a chemical step—a transvinylation reaction catalyzed by immobilized 10% Pd(OAc)_2_. The starting cinnamic acids (**119**), in the presence of sulfuric acid, were efficiently converted into the corresponding esters (**120**) with a residence time of 30 min at 60°C, achieving conversions of approximately 70%. The unreacted acids were recovered using a ‘catch‐and‐release’ protocol with an ion exchange resin (A21) at room temperature, enabling their reuse and further reducing the overall process cost of the procedure. The next step was the enzymatic reaction between the synthesized ester and tryptamine (**121**). The pure acyl‐transferase was immobilized onto glyoxyl‐agarose beads and packed into a PBR (1 mg_enzyme_·g^−1^_support_). Due to the poor solubility of the ester intermediates, toluene was used as the solvent. The optimal residence time was determined at 15 min at 28°C, while different flow rates were tested (around 0.1 mL·min^−1^). A membrane‐based separation followed this step to separate the product from any unreacted amines. The system ultimately produced the desired amides (**122**) on a gram scale through continuous operation over 24 h, indicating that multi‐gram production is feasible by increasing the reactor size.

**SCHEME 34:**
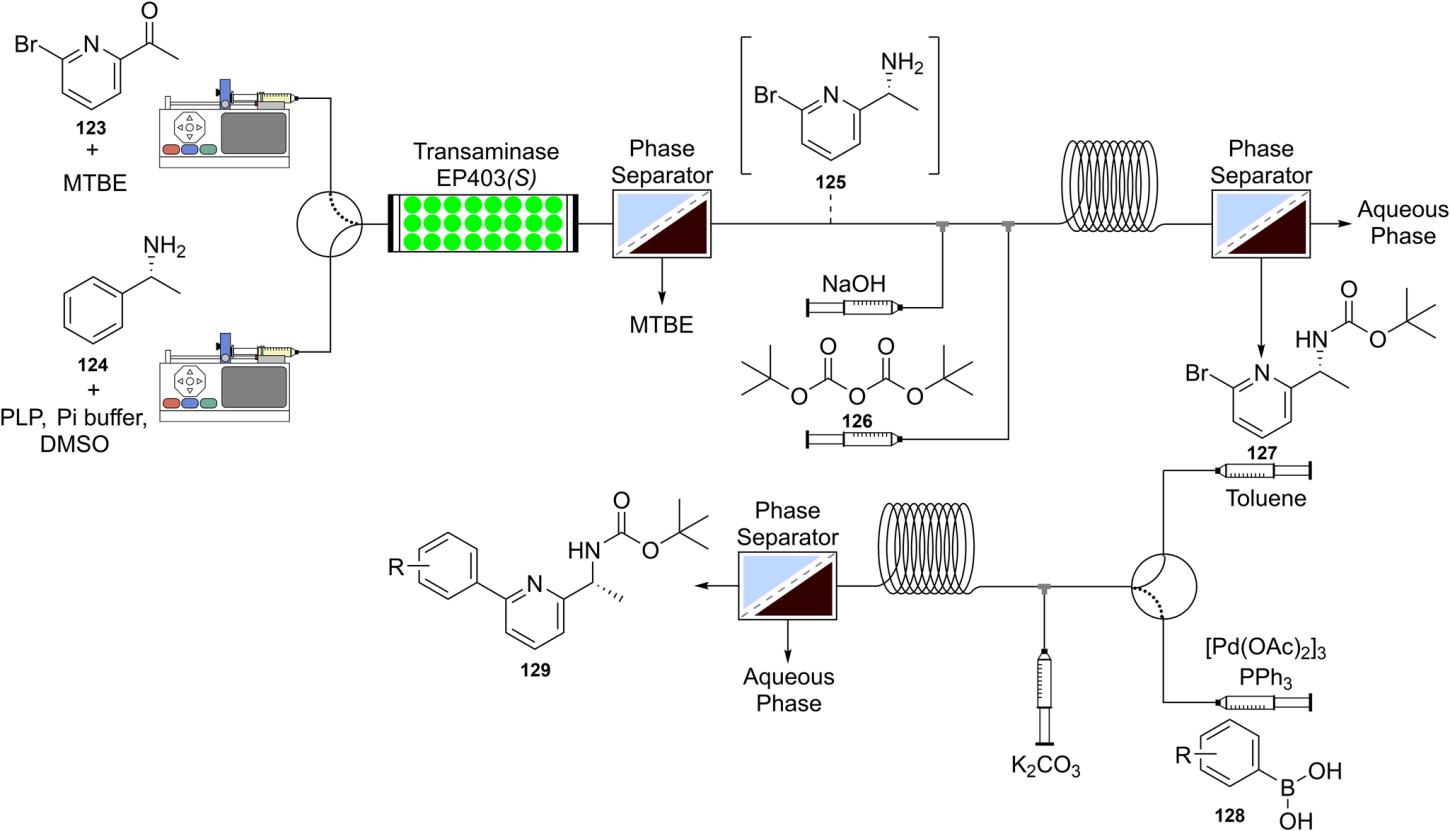
Continuous chemo‐enzymatic system for the synthesis of enantiopure biarylamines.

In 2024, Díaz‐Kruik et al. presented a multistep chemo‐enzymatic synthesis in continuous‐flow with a strategy of solvent switching by using phase separators (Scheme 34) [97]. Importantly, this work highlights how the integration of flow technologies, reaction engineering and a solvent‐switching systems can harness the complementary advantages of biocatalysis and organic synthesis in the streamlined production of pyridine‐aryl derivatives in three simple steps. The process consisted of a biocatalytic transamination performed by an *R*‐selective transaminase from *Thermomyces stellatus* (TsRTA) in a biphasic system, an inline N‐Boc‐protection step and, finally, a Suzuki–Miyaura cross‐coupling of the protected intermediate with a substituted phenylboronic acid (**128**). With this strategy, conversions up to 57% were achieved toward the carbamate as the final product, with a space‐time‐yield of 68 mg·L^−1^·h^−1^ and 99% enantiomeric excess. The process began with the biotransformation step, where 2.5 g of purified transaminase was immobilized using epoxy‐derivatized methacrylic resin (EP403/S) or a polysaccharide matrix (Agarose 6BCL) with an immobilization yield of >99%. The resulting transaminase loading was approximately 10 mg_protein_·g^−1^_support_ with specific activities of 12 and 7 U·g^−1^ on the two tested supports, respectively. The substrates, 2‐acetyl‐6‐bromopyridine (**123**) in MTBE and (*R*)‐methylbenzylamine (**124**) as the amine donor in phosphate buffer, were mixed in a 1:1 volume ratio. The reaction was carried out with a residence time of 20 min at 37°C, yielding 55% conversion and >99% ee, outperforming the batch process by 40 times in terms of productivity. After a phase separation, the organic phase was recirculated, while the aqueous phase moved to the second step. In the second step, 0.05 M of di‐*tert*‐butyl dicarbonate (**126**) in toluene and 0.2 M NaOH were added to the amine solution and the resulting mixture was directed to a 5 mL coil reactor maintained at 22°C for 30 min, reaching 97% conversion. A second phase separator then recovered the organic phase, which proceeded to the final step. For the last step, the Boc‐protected amine (**127**) in toluene was mixed with the substituted phenylboronic acid (**128**) in the presence of K_2_CO_3_ and directed into a 10 mL coil reactor maintained at 100°C for 5 min, enabling the cross‐coupling reaction. After this final reaction, phase separation yielded the desired product (**129**) in the organic phase with an isolated yield of 57% and >99% enantiomeric excess. Overall, the system ran for over 3 days, demonstrating considerable robustness. A key aspect of this study was the careful selection of the solvents for each step combined with the use of phase separators, which enable the seamless telescoping of intermediates. Furthermore, the application of flow chemistry mitigated potential hazards arising from the higher pressures required for the cross‐coupling reaction. By maintaining the system at 8 bar of pressure and 100°C in a relatively low‐volume coil reactor, the synthesis of these amines was safely achieved.

### 1.5 Isomerases

Isomerases have also been applied in industrial settings for a long time [98]. For instance, glucose isomerase has primarily been used to catalyze the interconversion of aldoses and ketoses, including the conversion of d‐glucose to d‐fructose, which is a key process in the sugar manufacturing industry. In the literature, a primary isomerase employed in continuous flow is hydroxylamine benzene mutase (HAB mutase). This enzyme performs a formal intramolecular transfer of hydroxyl groups, leading to the isomerization of hydroxylamines into aminophenols.

**SCHEME 35:**
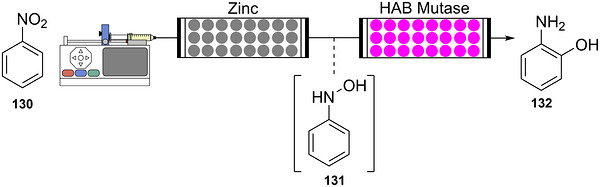
Setup of transformation of nitrobenzene (**130**) to ortho‐aminophenol (**132**).

In 2004, Luckarift et al. demonstrated a chemo‐enzymatic synthesis in a continuous‐flow system, in which a zinc‐catalyzed reduction of nitrobenzene (**130**) to the corresponding hydroxylaminobenzene (**131**) was followed by isomerization of the relatively unstable intermediate into ortho‐aminophenol (**132**) by HAB mutase B (Scheme 35) [99]. This represents an early example of a process combining chemical and enzymatic steps in continuous flow. The process consisted of two columns in series: the former containing zinc and the latter containing purified enzyme immobilized on biomimetically‐derived silica (biosilica). An aqueous solution of nitrobenzene (**130**) was pumped at a flow rate of 0.25 mL·min^−1^, resulting in the production of *ortho*‐aminophenol (**132**) with a conversion efficiency of 89% over a period of five hours. By doubling the flow rate, the researchers extended the system's running time to eight hours; however, the conversion efficiency dropped to 71%. As expected for such an early prototype, small quantities of hydroxyl‐aminobenzene (**131**) and aniline (a by‐product of the zinc reduction) were detected in the effluent, suggesting that hydroxyl‐aminobenzene concentration exceeded the biocatalytic capacity of the mutase column at higher flow rates. Finally, to explore the range of possible substrates, the researchers selected chloramphenicol. After experimentation, they found that only the HAB mutase A could catalyze this reaction toward the corresponding aminophenolic analog. Using a flow rate of 0.25 mL·min^−1^, they achieved 100% conversion efficiency for 24 h, with a product formation rate of approximately 0.2 mg·h^−1^·mg^−1^_total protein_. The process could be extended for an additional 24 h, simply by replacing the zinc cartridge, demonstrating a highly stable enzymatic step.

**SCHEME 36:**
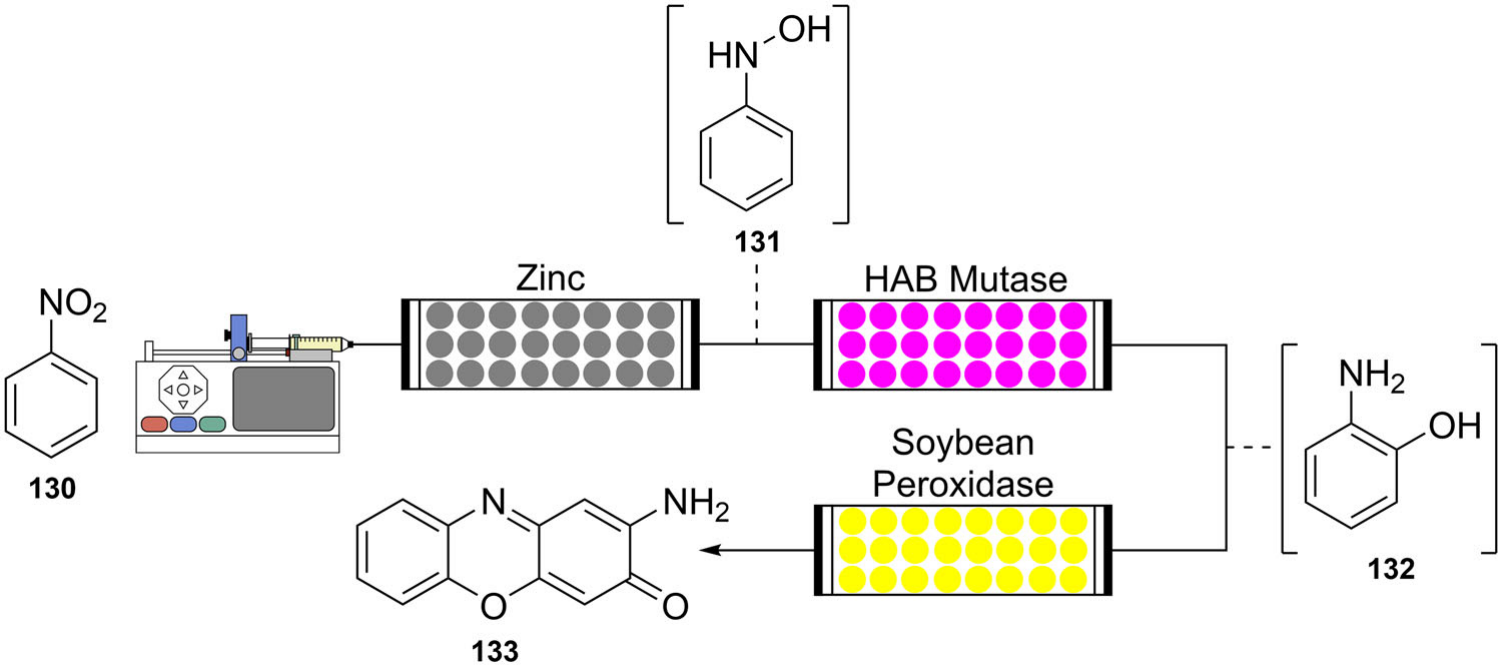
Setup of a multi‐step microfluidic process for the conversion of nitrobenzene (**130**) to 2‐aminophenoxazin‐3‐one (**133**).

In 2007, the same research group added a third step containing silica‐immobilized purified soybean peroxidase (SPB) to the previous setup (Scheme 36) [100]. This work represents one of the pioneering chemo‐enzymatic microfluidic reactor setups for the synthesis of a natural product from a commercially available starting material. Using three individual microfluidic chips in series, containing metallic zinc (mixed 1:1 with agarose beads), partially purified HAB mutase A, and soybean peroxidase, respectively—with the enzymes immobilized on silica—the researchers created a chemo‐enzymatic system for the synthesis of 2‐aminophenoxazin‐3‐one (**133**) (APO). For the silica‐immobilized SPB, a microfluidic channel was packed with an equal volume of agarose beads to achieve a final enzyme loading of 0.5 U. Starting from nitrobenzene (**130**), the first two reactions remained the same as before, followed by enzymatic oxidation and dimerization to APO (**133**) in the presence of H_2_O_2_ as an additional reagent. For the individual steps, the zinc chip gave a conversion of 25% at low flow rates, as aniline was again formed as a byproduct. However, in contrast to the biocatalyst‐containing chips, zinc chip's efficiency increased at higher flow rates. Therefore, the efficiency of the chemo‐enzymatic system had to be determined at lower flow rates, which were considered optimal for the biocatalytic steps. This led to a reduction in overall efficiency when the zinc chip was added in sequence. The enzyme‐containing chips could be washed and stored repeatedly at 4°C, retaining more than 50% of their original activity. For the complete synthesis, an aqueous solution of nitrobenzene (**130**) was introduced at a flow rate of 150 µL·h^−1^, which was significantly lower than in their previous work, resulting in an overall conversion efficiency of 19%. The ability of this system to convert a broad range of nitroarene substrates into their corresponding phenoxazinone products, combined with its versatile immobilization strategy, highlights its adaptability for diverse applications at the interface of biomacromolecules and chemical synthesis in flow.

## 2 Conclusion and Future Perspectives

### 2.1 Inherent Advantages That Define the Current State‐of‐the‐Art

Over the past two decades, chemoenzymatic synthesis under continuous‐flow conditions has evolved from being exploratory or proof‐of‐concept to being robust, flexible, and industrially relevant. The field draws heavily from and contributes to both biocatalysis (enzymology, protein engineering) and chemical engineering (reactor design, process intensification). Continuous‐flow reactors offer enhanced control over temperature, pressure, mixing, and residence time, which are critical parameters when working with more sensitive enzymes or hazardous or unstable intermediates. These features have empowered researchers to develop telescoped, multi‐step reaction sequences that are efficient, safe, and scalable. Table 1 summarizes the main advantages of continuous‐flow processing illustrated by the chemoenzymatic cascades discussed in the previous sections of this review, while Table 2 highlights aspects that still require further development to enable broader practical and industrial implementation.

**TABLE 1:** Overview of the main advantages of continuous‐flow processing compared with batch operation in chemoenzymatic cascades, illustrated by representative examples discussed in the schemes throughout this review.

Advantage of flow compared with batch	Cases
Safer handling of gases or operation under pressure.	Schemes 1, 5, 11, 26a and 28.
Higher reaction rates.	Schemes 1, 3, 6, 9, 10, 13, 14, 23a, 29a and 32.
Control of reaction rate to avoid formation of by‐products.	Schemes 3, 4, 9, 14, 16, 17, 18 and 19.
Integration of incompatible reaction steps.	Schemes 6, 8, 13, 17, 24, 26b, 27, 30 and 34.
Higher productivity or space‐time yield (STY).	Schemes 14, 15, 19, 24, 25, 29a, 31 and 34.
Safer handling of hazardous chemicals.	Schemes 5 and 10.
Easier work‐up and/or purification of crude product.	Schemes 4, 12, 14, 22 and 33.
Simple telescoping and compartmentalization.	Schemes 7, 20 and 21.

**TABLE 2:** Overview of key aspects that may require further optimization to enable broader practical and industrial implementation of chemoenzymatic cascades in continuous flow, illustrated by representative examples discussed in the schemes throughout this review.

Aspects requiring further development toward broader practical implementation	Cases
Moderate yield, recovery, or overall efficiency.	Schemes 1, 3, 4, 7, 8, 17, 27, 28, 34, 35 and 36.
Limited productivity, flow rate, space‐time yield (STY), or throughput.	Schemes 5, 6, 7, 8, 10, 28, 29a and 31.
Formation of side‐products or moderate diasteroselectivity (d.r.).	Schemes 7, 9 and 23.
Compatibility challenges between sequential steps.	Schemes 2, 3, 11, 17 and 30.
Scale‐up considerations (e.g., specialized equipment, scalability barriers, or handling of hazardous reagents).	Schemes 2, 3, 16 and 30.
Process complexity.	Schemes 24, 25 and 33.
Early‐stage or proof‐of‐concept demonstrations.	Schemes 12, 13, 14, 15, 20, 26a, 32, 34, 35 and 36.

**FIGURE 1:**
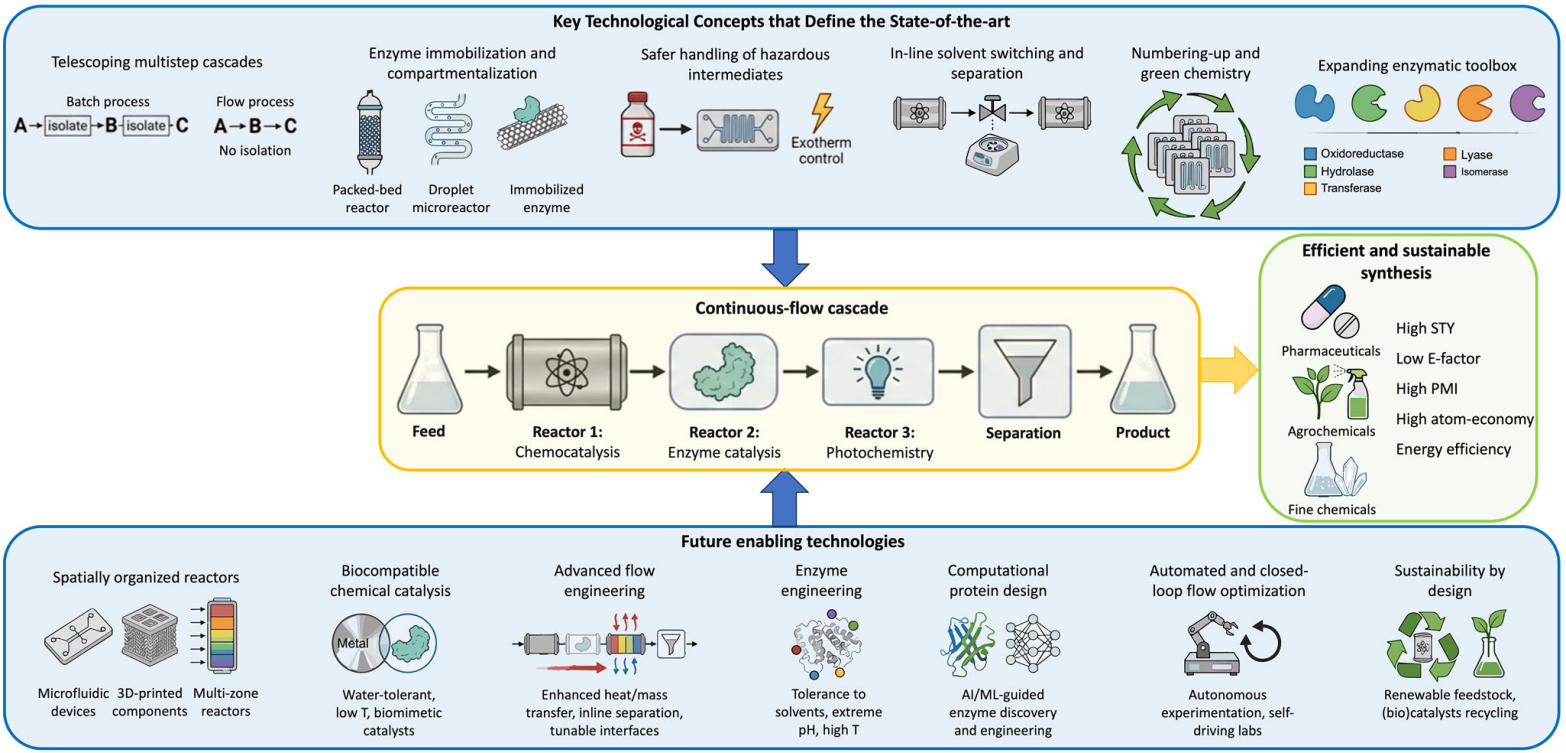
Conceptual overview of chemoenzymatic continuous‐flow synthesis. Continuous‐flow platforms enable the seamless integration of chemocatalysis, enzymatic catalysis and photochemical reactions through modular reactor architectures. Multistep integration, catalyst compartmentalization, reagent and solvent management, in‐line separations, and an expanded enzymatic toolbox enable the orchestration of complex chemoenzymatic cascades while improving safety, sustainability, and scalability. Emerging enabling technologies, including reactor engineering, biocompatible chemocatalyst design, enzyme engineering, computational protein design, and automated optimization are expected to further expand the scope and efficiency of chemoenzymatic flow synthesis.

In this section, we highlight key thematic advances that define the current state of the art, while the next section discusses future enabling technologies (Figure 1).

#### 2.1.1 Telescoping and Multistep Integration

One of the clearest advantages of flow systems is their ability to telescope multi‐step sequences with minimal intermediate handling. Early landmark examples include the continuous synthesis of 1‐monoacylglycerols from biodiesel‐derived glycerol [59], the synthesis of amino‐esters from bio‐derivable hydroxymethylfurfural [74], and the synthesis of chiral phenylethanol from acetophenone [55]—all using hydrolases—as well as the safe generation of O‐acetylcyanohydrins from HCN using lyases [60]; the latter case demonstrates the risk‐reduction potential of flow reactors when handling volatile and highly toxic reagents. Recent innovations have also enabled dynamic kinetic resolution (DKR) processes using temperature‐zoned packed‐bed columns combining chemical and enzymatic transformations in cascades, like in the resolution of N‐Boc‐phenylalanine ethyl thioester [61]. Temperature‐zone‐controlled approaches have also been used for the synthesis of carbamates from alcohols and amines [73].

Another example was the integration of an enzymatic transketolase step with a Diels–Alder cycloaddition [94], or the combination of enzymatic decarboxylation with Pd‐catalyzed cross‐coupling [84], which exemplify the potential of hybrid catalytic systems under flow conditions. Zinc‐catalyzed reduction of nitrobenzene, with a subsequent enzymatic isomerization of hydroxylaminobenzene to ortho‐aminophenol using a mutase enzyme, demonstrates the advantage of cascades in flow reactors when unstable intermediates are generated [99].

In more complex examples, the combination of whole‐cell biotransformations for steroid functional group manipulation (i.e., asymmetric reduction of ketone to alcohol) with chemical derivatization (i.e., acylation) facilitates final product isolation without any intermediate or final purification step, while achieving STY one order of magnitude higher than in batch [80]. Finally, biocatalytic reactions have been exploited to tag impurities formed after a chemical reaction to facilitate the isolation of the desired product in pure form—two steps that were streamline‐integrated in a two‐reactor flow setup [68].

#### 2.1.2 Safety and Process Intensification

Continuous flow technology inherently supports safer and more compact reaction setups. Controlled stoichiometry and rapid mixing in microfluidic modules not only prevent runaway reactions but also enable real‐time control over sensitive chemical, chemo‐catalytic, or enzymatic steps. For hazardous reagents like peracids or hydrogen cyanide, in situ generation and immediate consumption by the enzyme within a closed loop significantly enhance operator safety and reduce waste. Besides the abovementioned generation of O‐acetylcyanohydrins catalyzed by a lyase from an aldehyde and HCN [60], the development of in situ peracid generation for Baeyer–Villiger oxidations via lipase promiscuity is another notable example [65]. This latter example also highlights the creative repurposing of common enzymes for novel reactivity under flow.

#### 2.1.3 Compartmentalization and Reactor Engineering

Compartmentalization strategies have evolved to manage (bio)catalyst incompatibility and reaction orthogonality in cascades. Approaches include droplet‐based Pickering emulsions (e.g., synthesis of O‐acylated cyanohydrin) [71], immobilized enzyme beds, and 3D‐printed modular flow units, all of which mimic cellular compartmentalization and allow fine spatial control over catalyst localization. Microreactor technology permits better control of reaction conditions, thus enhancing selectivity in sensitive processes such as the synthesis of polymers with controlled molecular weight and dispersity [69]. Support materials such as multi‐walled carbon nanotubes [65], gentle packing agents, and functionalized polymers have improved enzyme reusability, reduced clogging, and prolonged operational lifetimes. Modular systems are also increasingly combined with real‐time monitoring and feedback loops, moving toward “smart” flow chemistry systems.

#### 2.1.4 Systems Engineering to Overcome Solvent and Byproduct Incompatibilities

Solvent choice is critical as it influences substrate solubility and downstream processing. Enzymes can operate in biphasic systems, nonconventional solvents such as organic solvents, ionic liquids, or even in neat substrates. For instance, solvent‐free approaches were discussed for the chemo‐enzymatic synthesis of chiral β‐amino esters and β‐amino amides by combining a thermal Aza‐Michael addition with a lipase‐catalyzed amidation [58], or for the dynamic kinetic resolution of amines using a Pd‐catalyst and, again, a lipase‐catalyzed amidation [64]. In another example, dimethyl carbonate has been used both as a solvent and a reagent, simplifying reaction schemes and boosting atom economy [63]. In all these cases, the neat substrate reaction conditions were compatible between the chemical and enzymatic steps. Another illustrative example was the chemo‐enzymatic tandem synthesis of (E)‐4‐hydroxy stilbene, where a deep eutectic solvent (DES)/water mixture was employed to enable both an enzymatic decarboxylation and a subsequent Heck reaction, demonstrating excellent compatibility between the two steps under such unconventional solvent conditions [84]. However, finding solvents that are compatible with both chemical and enzymatic steps is not always possible, but flow systems allow circumventing this problem. Recent works discussed in this review have introduced in‐line solvent‐switching strategies, allowing enzymes and chemical catalysts with incompatible solvent preferences to operate sequentially without intermediate isolation. This is exemplified by the streamlined production of pyridine‐aryl derivatives from 2‐acetyl‐6‐bromopyridine in three simple steps [97], or by the production of 5‐hydroxymethylfurfural(HMF)‐derived plasticizers from HMF [67]. Similarly, in‐line separation units enable the removal of a byproduct from a preceding enzymatic reaction (e.g., ethanol generated from the enantioselective amidation of an amine with an ester), thereby enabling DKR processes that would otherwise be impossible in batch [72]. Finally, we presented an example where incompatible biocatalytic and photocatalytic steps were successfully integrated in continuous flow, overcoming cross‐inhibition issues. The system design also ensured uniform irradiation during the photocatalytic step, preventing product decomposition. While such combinations remain rare, they are expected to grow significantly in the future [69].

#### 2.1.5 Sustainability and Scalability

Flow biocatalysis aligns naturally with green chemistry principles: it limits the use of stoichiometric reagents and reduces waste through telescoping. Moreover, the numbering‐up strategy—parallel operation of identical microreactors—offers an elegant solution to scalability, bypassing the limitations of traditional batch scale‐up. For instance, the chemoenzymatic synthesis of hordenine from l‐tyrosine using a tyrosine decarboxylase followed by reductive alkylation achieved an E‐factor of 36 using pic‐BH_3_ suspended in celite, highlighting the sustainability of optimized hybrid flow systems [85]. Similarly, the flow systems for the synthesis of procainamide and procaine using an acyltransferase in pure organic solvent were fast, robust, and scalable using commercially available packed‐bed reactors, while maintaining enzyme stability and reusability [95].

#### 2.1.6 Enzyme Class Diversity and Emerging Directions

While hydrolases, especially lipases, dominated early industrial implementations due to their robustness and availability, oxidoreductases, transferases, lyases, and isomerases have increasingly proven viable under continuous flow conditions. From C─C and C─N bond formation to stereoselective rearrangements and asymmetric reductions, these systems now span a broad synthetic repertoire.

For example, the use of immobilized horseradish peroxidase in a fully automated flow setup enabled the synthesis of the natural product Grossamide, illustrating the potential of the peroxidase subclass of oxidoreductases [76]. The four‐step chemoenzymatic route for Captopril, despite lacking full integration of the enzymatic and chemical steps, demonstrates the applicability of the oxidase subclass of oxidoreductases and is notable for its approach to oxygen supply for the regeneration of the resting state of the enzyme [82]. Other efforts expanded the scope to the dehydrogenase subclass of the oxidoreductases to accomplish the deracemization of alcohols via integrated heterogeneous chemo‐enzymatic catalysis under fixed‐bed flow [78], or the enantioselective synthesis of chiral α‐mono‐ and di‐fluoromethyl amines [81], or the asymmetric organo‐biocatalyzed synthesis of chiral 1,3‐diols [79], and offer a promising strategy for accessing novel scaffolds otherwise difficult to obtain. For these NAD(P)H‐dependent enzymes, flow setups can already enable continuous cofactor regeneration via catch‐and‐release ion exchange resins [13], while in the future, the implementation of electrochemical or photoredox strategies for in‐situ cofactor regeneration could also be realized. Notably, innovations in cofactor recycling cut costs and extend the applicability of oxidoreductases in long‐term operations.

Other underexplored enzyme classes, such as dehydratases, are also being introduced, as exemplified by the gold‐catalyzed oxidation followed by enzymatic dehydration to access 2‐keto‐3‐deoxy sugar acids, also demonstrating potential for expanding structural diversity via enzyme promiscuity [87]. Even the more rarely used class of isomerases has demonstrated feasibility, such as in the flow‐based isomerization of hydroxylaminobenzene to ortho‐aminophenol, which was coupled with upstream and downstream steps in a single microfluidic reactor; this process was even extended to the synthesis of a natural product by introducing a peroxidase in a third, consecutive reactor [100].

### 2.2 Future Perspectives

The field of chemoenzymatic continuous‐flow synthesis is progressively evolving from predominantly academic laboratory demonstrations toward a broader role in modern synthetic chemistry. Future innovations will fundamentally reshape how we design, scale, and control cascade reactions that blend the best of biocatalysis and chemo‐catalysis. This section highlights main areas for future innovation.

#### 2.2.1 Compartmentalized Reactors, Spatial Organization, and Dynamic Regulation for Orthogonal Catalysis

The future of chemoenzymatic continuous flow lies in modular and spatially organized reactor systems [101]. Innovations such as microfluidic devices [102], 3D‐printed components [103, 104], and multi‐zone flow reactors will allow truly orthogonal catalytic steps—previously incompatible in batch—to function seamlessly in sequence. These designs offer precise control over local conditions, enabling the orchestration of complex cascades with minimal work‐up, solvent exchange, or intermediate handling.

Co‐immobilization of enzyme cascades has emerged as a powerful strategy to improve stability and throughput [25, 53, 105]. Strategic placement of multiple enzymes on solid supports or within microchannels enables substrate channeling, balances pathway kinetics, and minimizes diffusion losses. Recent studies have demonstrated dramatic increases in space‐time yields through such architectures, underscoring their potential for industrial implementation [106, 107].

Beyond co‐localization, physical separation of catalytic steps via droplets, parallel reactors, or semi‐permeable membranes allows for fine‐grained control over cascade performance. Meanwhile, dynamic systems—where enzyme activity can be modulated in real‐time via light, temperature, or pH—offer a path to adaptive, self‐regulating flow processes that respond to changing reaction conditions on the fly.

#### 2.2.2 Redesigning Chemical Catalysts for Biocompatibility

To support this integration, chemical catalysts will need to evolve. Water‐tolerant, low‐temperature, and biomimetic catalysts are under development to ensure better compatibility with biocatalysts. These next‐generation systems will minimize side reactions, reduce the need for protective group strategies, and streamline the overall synthesis workflow [108].

#### 2.2.3 Flow Reactor Engineering

Continued advances in reactor engineering will expand the operational window of chemoenzymatic cascades. Flow systems with enhanced heat/mass transfer, in‐line separators, and tunable interfaces will support longer and more diverse reaction sequences. These capabilities will enable reactions that remain balanced, efficient, and scalable even at high throughput [31].

#### 2.2.4 Enzyme Engineering

Natural enzymes often lack the required robustness, selectivity, or compatibility for continuous operation. Enzyme engineering—via directed evolution, site‐directed mutagenesis, and de novo design—is now delivering catalysts tailored for flow. Enzymes with high tolerance to solvents, extreme pH, or elevated temperature will expand the applicability of chemo‐biocatalysis across a wider synthetic space [44, 45, 46, 47, 48, 49, 50, 51, 52].

#### 2.2.5 Computational Protein Design and Machine Learning

Machine learning (ML) is revolutionizing enzyme optimization and evolution [48, 109]. ML‐guided campaigns can identify beneficial mutations far more efficiently than traditional methods, reducing experimental burden while improving performance [110, 111]. Deep‐learning models may soon design novel biocatalysts and enzyme scaffolds for previously inaccessible transformations. In the future, enzymes could be designed not only for inherent activity or stability, but also for compatibility within complex multi‐catalytic environments.

#### 2.2.6 Automation and Closed‐Loop Optimization

Automated flow platforms, powered by ML and AI, are set to eliminate many of the bottlenecks in process development. Smart algorithms can optimize reagent ratios, flow rates, residence times, and cascade ordering using minimal experimental input [112]. Closed‐loop systems will enable continuous improvement via autonomous experimentation, allowing “self‐driving labs” to develop and optimize chemoenzymatic cascades around the clock [113, 114, 115].

#### 2.2.7 Sustainability by Design

Sustainability is not an afterthought but a primary driver of this field. Future systems will integrate renewable feedstocks, enzyme and solvent recycling, and dynamic resource monitoring. At the same time, the development and adoption of standardized metrics for evaluating process performance and sustainability will be essential to enable meaningful comparisons between different systems, particularly in the context of process intensification. Metrics such as space–time yield (STY), E‐factor, process mass intensity (PMI) and atom economy should be reported in a consistent manner across studies. Life‐cycle assessment and energy‐efficiency metrics should become standard tools in early development, ensuring that new processes meet the demands of both performance and ecological responsibility [116].

## 3 Concluding Remarks and Critical Assessment

In this review, we have analyzed 36 examples of chemoenzymatic processes in continuous flow spanning different stages of development, from early proof‐of‐concept studies to robust experimental demonstrations and systems with clear potential for industrial implementation. In general, these chemoenzymatic processes allow better yields and selectivity than the analogous batch processes, while also being greener and safer. At the moment, the systems that appear most promising for further industrial development are those with relatively simpler reactor configurations, fewer steps, and high space‐time yields. Notably, high selectivity, mild reaction conditions, and waste reduction are typically enabled by the introduction of enzymes in these chemoenzymatic processes.

The factors that are currently preventing a quicker uptake of this technology by the chemical industry are: i) the substantial capital investment required for transitioning from established batch processes to flow‐based manufacturing (e.g., purchase of new equipment, adaptation of existing infrastructure); ii) the required steps of optimization and validation of the new process in continuous flow; iii) the associated risk of abandoning consolidated manufacturing operations; and iv) regulatory frameworks that typically favor existing processes, which are operated in batch. Consequently, many industries still heavily rely on well‐established batch technologies.

The most immediate opportunities therefore lie in the transition from batch to continuous‐flow operation for processes that are currently performed in batch under unsafe or inefficient conditions. In these cases, the advantages associated with continuous‐flow manufacturing outweigh the costs and risk factors outlined above. However, regulatory pressure toward greener and more sustainable chemical processes is expected to increase in the future, thereby making the broader adoption of both biocatalysis and continuous‐flow operation increasingly important for the chemical industry. Under these conditions, chemoenzymatic flow systems could emerge as valuable platforms for more efficient, versatile, and sustainable chemical manufacturing.

To conclude, taken together, these innovations could lead to a paradigm shift in synthesis. The seamless integration of chemo‐ and biocatalysis under continuous flow—further enhanced by automation, artificial intelligence, and advances in materials and enzyme engineering—has the potential to redefine how complex molecules are produced. As these enabling technologies converge, we can anticipate fully integrated, end‐to‐end cascades capable of producing pharmaceuticals, agrochemicals, and fine chemicals with high efficiency and sustainability. Chemoenzymatic continuous flow processes are poised to become a cornerstone of 21st‐century chemical synthesis.

## Conflicts of Interest

The authors declare no conflicts of interest.

## Data Availability

The data that support the findings of this study are available from the corresponding author upon reasonable request.
